# Pharmacological inhibition of ENT1 enhances the impact of specific dietary fats on energy metabolism gene expression

**DOI:** 10.1073/pnas.2321874121

**Published:** 2024-08-29

**Authors:** Erwann Pain, Stuart Snowden, Joseph Oddy, Sonia Shinhmar, Yousef M. A. Alhammad, Jason S. King, Annette Müller-Taubenberger, Robin S. B. Williams

**Affiliations:** ^a^Centre for Biomedical Sciences, Department of Biological Sciences, School of Life Sciences and the Environment, Royal Holloway University of London, Egham TW20 OEX, United Kingdom; ^b^Department of Cell Physiology and Metabolism, Faculty of Medicine, University of Geneva, Geneva 4 CH-1211, Switzerland; ^c^Department of Biomedical Sciences, University of Sheffield, Sheffield S10 2TN, United Kingdom; ^d^Department of Cell Biology, Biomedical Center, Ludwig Maximilian University of Munich, Planegg-Martinsried 82152, Germany

**Keywords:** decanoic acid, *Dictyostelium discoideum*, ketogenic diets, medium chain triglycerides, energy metabolism

## Abstract

Medium chain fatty acids are commonly consumed as part of diets for endurance sports and as medical treatment in ketogenic diets where these diets regulate energy metabolism and increase adenosine levels. However, the role of the equilibrative nucleoside transporter 1 (ENT1), which is responsible for adenosine transport across membranes in this process, is not well understood. Here, we investigate ENT1 activity in controlling the effects of two dietary medium chain fatty acids (decanoic and octanoic acid), employing the tractable model system *Dictyostelium.* We show that genetic ablation of three ENT1 orthologues unexpectedly improves cell proliferation specifically following decanoic acid treatment. This effect is not caused by increased adenosine levels triggered by both fatty acids in the presence of ENT1 activity. Instead, we show that decanoic acid increases expression of energy-related genes relevant for fatty acid β-oxidation, and that pharmacological inhibition of ENT1 activity leads to an enhanced effect of decanoic acid to increase expression of tricarboxylicacid cycle and oxidative phosphorylation components. Importantly, similar transcriptional changes have been shown in the rat hippocampus during ketogenic diet treatment. We validated these changes by showing enhanced mitochondria load and reduced lipid droplets. Thus, our data show that ENT1 regulates the medium chain fatty acid-induced increase in cellular adenosine levels and the decanoic acid-induced expression of important metabolic enzymes in energy provision, identifying a key role for ENT1 proteins in metabolic effects of medium chain fatty acids.

## Significance

Medium chain fatty acids are used as supplements in therapeutic roles related to ketogenic diets and for high-intensity exercise, where they regulate energy metabolism. As energy metabolism is based on adenosine-containing chemicals, we investigate a role for adenosine transport by equilibrative nucleoside transporter 1 (ENT1) in regulating the effects of these fatty acids. Using a tractable model system, we show that decanoic acid increases adenosine levels and enhances energy-related gene expression. We further show that pharmacological inhibition of ENT1 enhances expression of energy-related genes following decanoic acid treatment and increases mitochondrial load and fatty acid metabolism. Thus, we provide insight into how specific medium chain fatty acids improve cellular energy processes, and that this effect can be enhanced by pharmacological inhibition of ENT1 activity.

Fatty acids are one of the three macronutrients that play a central role in physiology and metabolism (1). Medium chain fatty acids, defined as 6 to 12 carbons in length, are widely used as a supplement for endurance sports (2, 3), where they provide enhanced energy. In addition, medium chain fatty acids are prescribed either as an additive to a “classical” ketogenic diet (4) or in the medium chain triglyceride (MCT) ketogenic diet (5). Both these ketogenic diets are used as a treatment for drug-resistant epilepsy (6–8), but are increasingly being used for the treatment of other disorders (9, 10). The antiseizure properties of ketogenic diets have long been believed to be due to the action of ketone bodies, that are three fatty acid metabolites formed during ß-oxidation (11). However, some studies have suggested blood ketone levels do not correlate with seizure control (12, 13), and ketones do not directly block seizure activity (14). Despite this, a range of ketone-related mechanisms are still proposed in the treatment of epilepsy (15, 16), including metabolic changes induced by ketones as an energy source to provide energy homeostasis. Finally, the recent discovery of specific medium chain fats, that are produced by hydrolysis of MCTs in the gut, in particular decanoic acid, provides specific antiseizure effects independent of ketone formation, such as direct α-amino-3-hydroxy-5-methyl-4-isoxazolepropionic acid (AMPA) receptor inhibition (14, 17), mammalian target of rapamycin complex 1 (mTORC1) inhibition (18), and peroxisome proliferator-activated receptor gamma (PPARγ) activation (19, 20) to increase mitochondrial biogenesis (21) and cellular energy, with these mechanisms leading to seizure control (5).

Adenosine-based signaling represents the primordial mechanism for energy transfer and shows strong conservation throughout evolution (22). Adenosine levels increase in the brain during ketogenic diet treatment (23–25) to activate the adenosine 1 receptor (A1R) and reduce synaptic excitability (25–28). Movement of adenosine into and out of cells is regulated by the equilibrative nucleoside transporter 1 (ENT1) (29) which is overexpressed in epileptic patients and mice during epileptogenesis, to reduce extracellular adenosine and decrease A1R-depedent synaptic inhibition (30, 31). However, our understanding of how ketogenic diets regulate these effects, and the potential for alterations in ENT1 activity to modify adenosine signaling and cell function remains to be explored.

Decanoic acid was identified as a potential seizure control treatment initially in the model system *Dictyostelium discoideum* through the regulation of phosphoinositide signaling (32, 33). The model has also been used to identify an effect of decanoic acid on diacylglycerol signaling potentially relating to the treatment of bipolar disorder (34), and in the inhibition of mTORC1 (18). These discoveries led to the successful development of a new dietary treatment for drug-resistant epilepsy (35), and a range of novel drug-like compounds with efficacy in seizure control (33, 36, 37). The use of *D. discoideum* in research provides a range of advantages including a genome similar to higher eukaryotes (38), but with the ability to rapidly ablate single or multiple genes in isogenic cell lines (39), and that resulting discoveries in *D. discoideum* have been translated into preclinical models (40). Importantly, the role of bioactive compounds, such as decanoic and octanoic acid, can be investigated in this model using changes in cell proliferation as a bioassay for activity, where isogenic mutants can be assessed for loss or gain of sensitivity to either fatty acid (18, 32–34).

In this paper, we investigate the bioactivity of decanoic and octanoic acid as regulated by ENT1 proteins using *D. discoideum*. We show that loss of multiple *ent1* genes in this model results in an unexpected effect of increased cell proliferation within a defined window of decanoic acid concentration, not shown for octanoic acid. Investigating this effect identified that both decanoic and octanoic acid increase adenosine levels, in the presence of ENT1 activity, which is therefore unlikely to underlie this enhanced proliferation response. The functions of the *D. discoideum* ENT1 proteins are likely to be conserved since expression of the human protein complements the loss of endogenous ENT1 proteins to restore wild type (WT) response to decanoic acid. We further show that decanoic acid treatment triggers an increase in the expression of energy metabolism-related genes, including β-oxidation of fatty acids, and tricarboxylicacid (TCA) cycle and oxidative phosphorylation-related genes that were more broadly increased following inhibition of ENT1 activity. These effects were validated by identifying increased mitochondria volume and number, and reduced lipid droplet volume and number following decanoic acid treatment in combination with ENT1 inhibition (ENT1i). These studies thus provide a key insight into the regulation of cellular energy levels by medium chain fatty acids.

## Results

### Bioactivity of Decanoic Acid Is Regulated by ENT1 Orthologues.

**Fig. 1.**
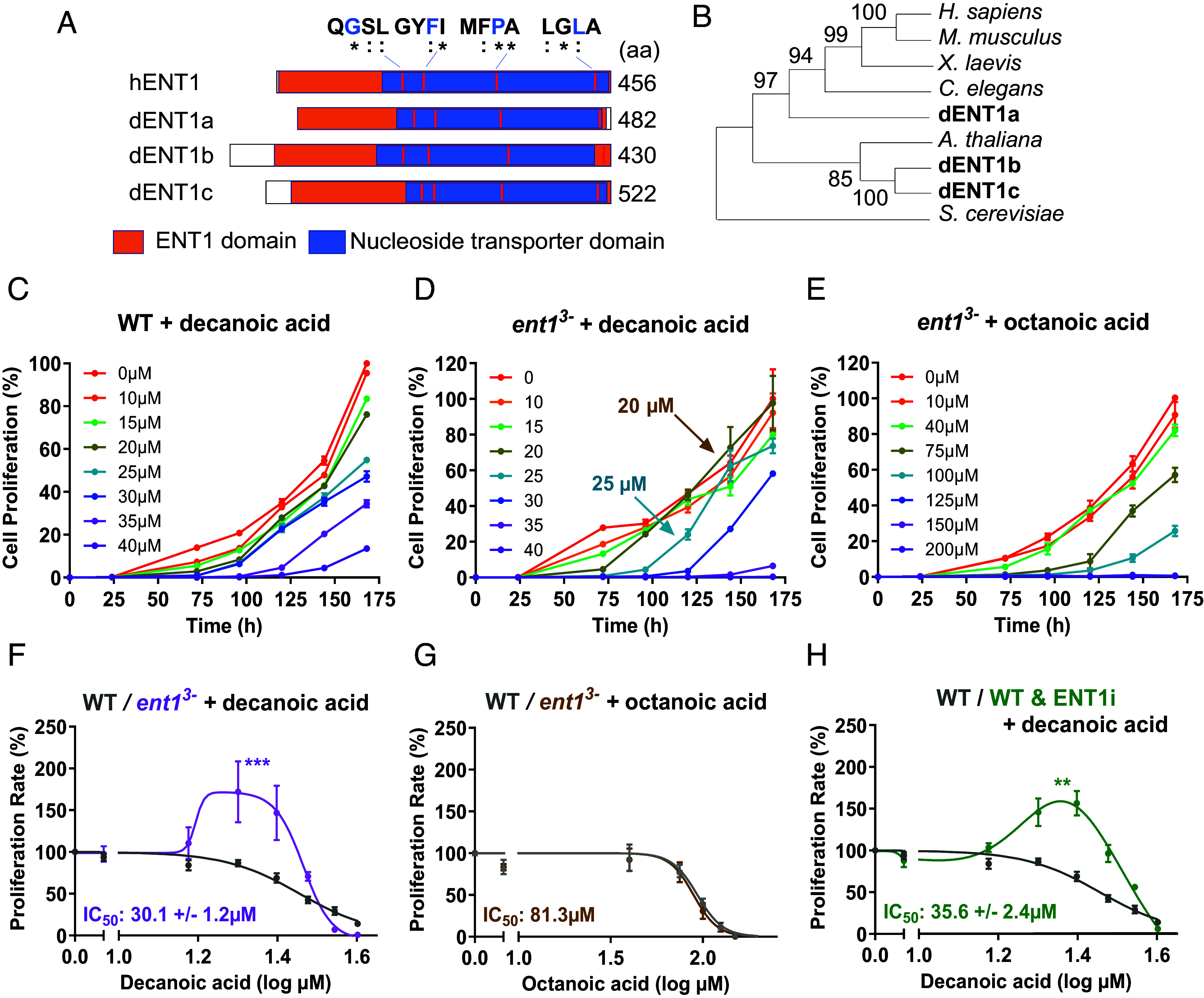
*D. discoideum* contains three human ENT1 orthologues that regulate cell proliferation sensitivity to decanoic acid and adenosine levels. (*A*) Human (Uniprot Q99808) and *D. discoideum* ENT1 proteins (dENT1a, DDB_G0281513, Uniprot Q54TT3; dENT1b DDB_G0283439, Uniprot Q54R17; and dENT1c, DDB_G0281515, Uniprot Q54TT2). Domain structure analysis shows a nucleoside transporter domain, present in all nucleoside transporters, and an ENT1 domain specific to passive nucleoside transporters of similar size (amino acids), with conservation of catalytic amino acid (in blue) (“*” indicating identical amino acids in the ENT1 proteins; “:” indicating amino acids with similar structures and functions in the ENT1 proteins (*SI Appendix*, Fig. S1). (*B*) Phylogenetic analysis of ENT1 proteins from multiple organisms, where numbers represent the probability of an evolutionary branch separation. (*C*–*H*) Analysis of medium chain fatty acid effects on cell proliferation in (*C*) WT cells treated with decanoic acid show a dose-dependent reduction in cell proliferation; (*D*) *ent1^3−^* cells treated with decanoic acid provide an unexpected increase in cells in proliferation at specific concentrations (20 to 25 µM); (*E*) *ent1^3−^* *ent^3−^* cells treated with octanoic acid provide a dose-dependent reduction in cell proliferation. To quantify these effects, secondary plots comparing proliferation rate (120 to 144 h) in WT and *ent1^3−^* cells for (*F*) decanoic acid treatment, (*G*) octanoic acid treatment, or in (*H)* WT cells with decanoic acid treatment in the presence of an ENT1 inhibitor (ENT1i, 10 µM 6-S-[(4-nitrophenyl)methyl]-6-thioinosine (NBTI)) with statistical analysis using a two-tailed Mann–Whitney test (*P = 0.05; **P < 0.01; ***P < 0.001).

Bioinformatics analysis of the *D. discoideum* genome identified three potential orthologues of the human ENT1 protein (hENT1), termed dENT1a, dENT1b, and dENT1c (Fig. 1*A*). Primary sequence alignment showed high conservation between *D. discoideum* and human proteins with 70% (dENT1a), 55% (dENT1b), and 53% (dENT1c) similarity (*SI Appendix*, Fig. S1), with distinct expression profiles during development (*SI Appendix*, Fig. S2). In addition, the proteins were of a similar size, with conserved essential amino acids required for adenosine transport (G179, F209, P308, and L442) suggesting a similar function between *D. discoideum* and hENT1 proteins (41–43). Cladistic analysis indicates that dENT1a is more closely related to animal kingdom ENT1 proteins than dENT1b and dENT1c (Fig. 1*B*).

To investigate a role for *D. discoideum* ENT1 proteins in response to decanoic and octanoic acids, various mutants were produced containing single (*SI Appendix*, Figs. S3 and S4), double, and triple ENT1 knockouts, and sensitivity of these mutants was investigated following fatty acid treatment. In these experiments WT or mutant cells were treated with decanoic or octanoic acid at a range of physiologically relevant concentrations (35) over 7 d, and cell proliferation was monitored. In WT cells, decanoic acid treatment provided dose-dependent reduction in cell proliferation (Fig. 1*C*; IC_50_ 29.02 ± 1.18 µM), with only small changes in IC_50_ values for ablation of individual *ent1a*, *ent1b,* and *ent1c* genes (*SI Appendix*, Fig. S5). However, ablation of two *ent1* genes (in *ent1a-b*^−^, *ent1b-c^−^*) or all three *ent1* genes (in *ent1*^3−^) provided an unexpected increase in cell proliferation at a specific concentration range of decanoic aid (20 to 25 µM) (Fig. 1*D* and *SI Appendix*, Fig. S6). The growth rate of WT cells treated with 20 µM decanoic acid decreased to 90.2% of solvent-only control cells, but significantly increased in *ent1*^3−^ cells to 172% (*P* = 0.007). This effect was specific to decanoic acid, since octanoic acid treatment did not increase in cell proliferation at any concentration (Fig. 1*E* and *SI Appendix*, Fig. S7). Analysis of the rate of cell proliferation at increasing concentrations of decanoic acid or octanoic acid confirmed this increase with decanoic acid treatment only (Fig. 1 *F* and *G*). This increase was likely caused by loss of ENT1 function, since treatment of WT cells with a specific ENT1 inhibitor, NBTI [6-*S*-[(4-nitrophenyl)methyl]-6-thioinosine] (44) reproduced this increase in growth rate to 163.6% (*P* < 0.001) at 20 µM decanoic acid (Fig. 1*H*). Thus, these data suggest that *D. discoideum* ENT1 proteins play a partially redundant role, and loss of ENT1 activity identifies an unusual improvement in cell proliferation in response to decanoic acid at a defined concentration range following extended treatment.

### Decanoic Acids Elevates Intracellular Adenosine Levels through Adenosine Transport.

**Fig. 2.**
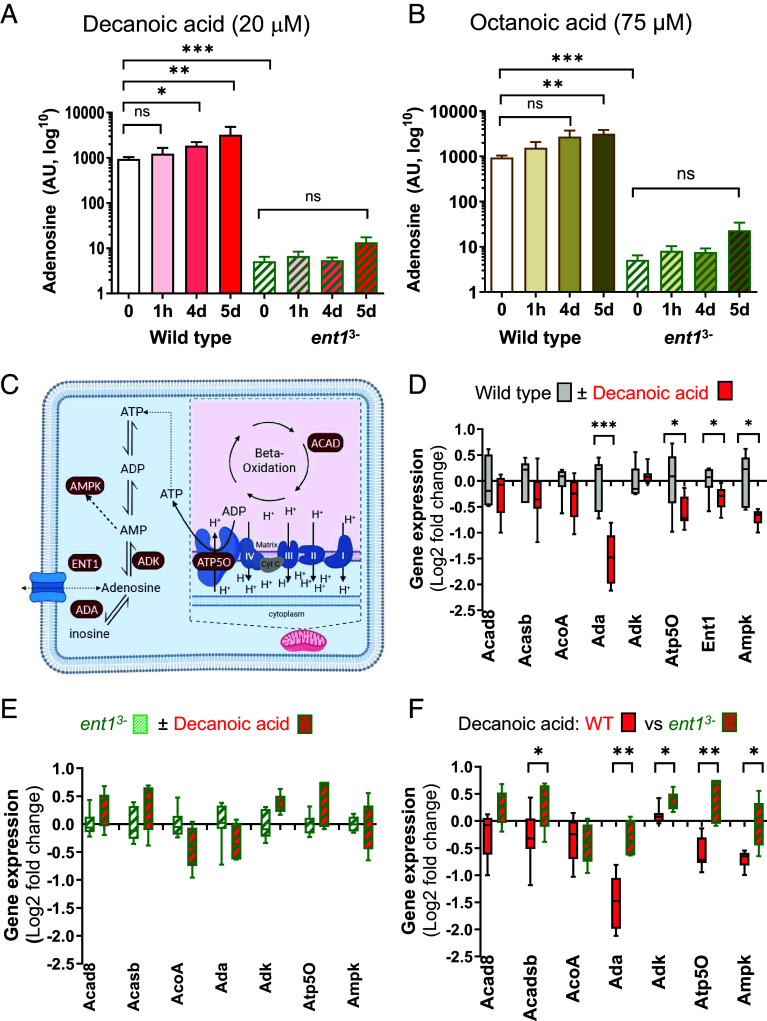
Decanoic acid and octanoic acid elevate adenosine levels through ENT1s but only decanoic acid regulates energy metabolism in *D. discoideum *dependent upon ENT1 activity**. *D. discoideum* WT or *ent1^3−^* cells were grown (*A*) with or without decanoic acid (20 µM) treatment, or (*B*) with or without octanoic acid (75 µM) treatment for indicated time periods (“h” hours or “d” days), and intracellular adenosine levels were assessed by LC–MS. (*C*) Adenosine signaling schematic indicating key roles of *ACAD8* (isobutyryl-CoA dehydrogenase, DDB_G0288647) that catalyzes the first dehydrogenation step in the β-oxidation of fatty acyl-CoA derivatives, *ACASB* (acyl-CoA dehydrogenases, DDB_G0282967), and *ACOA* (acyl-CoA oxidase, DDB_G0283261) involved in fatty acid metabolism, *ADA* (DDB_G0287371) which catalyzes the irreversible deamination of adenosine to inosine, *ADK* (adenosine kinase, DDB_G0286057) that phosphorylates adenosine into AMP, *ATP50* (F-type H^+^-transporting ATPase subunit O, DDB_G0283283) part of the mitochondria membrane ATP synthase involved in ATP production, *ENT1a* (ENT1, DDB_G0283439) in plasma and mitochondrial membranes involved in movement of adenosine across membranes, and *AMPK* (5′ AMP-activated protein kinase, DDB_G0272542) involved in regulating cell energy status by altered ratio of AMP/ATP. Gene expression changes, measured by qPCR, were compared in (*D*) WT cells and (*E*) *ent1^3−^* cells with or without 20 µM decanoic acid treatment for 4 d, and (*F*) WT and cells with or without decanoic acid treatment. All data are derived from three independent experiments in triplicate, with statistical analysis using a two-tailed Mann–Whitney test, *P < 0.05; **P < 0.01; ***P < 0.001.

Since decanoic acid treatment caused an unexpected improvement of cell proliferation in cells lacking ENT1 function, cellular levels of adenosine were determined in WT and *ent1*^3−^ cells following decanoic and octanoic acid treatment. In these experiments, WT and *ent1*^3−^ cells were incubated for 1 h, 4 or 5 d to account for both acute and chronic effects with either 20 µM decanoic or 75 µM octanoic acid (providing the same inhibitory effect on WT cells as 20 µM decanoic acid (*SI Appendix*, Fig. S6), and intracellular adenosine levels were measured using liquid chromatography–mass spectrometry (LC–MS). In WT cells, acute treatment with decanoic acid (1 h) induced a nonsignificant increase in adenosine level by 31%, while chronic treatment increased adenosine levels by 96% at 4 d (*P* = 0.03) and 245% at 5 d (*P* = 0.004, Fig. 2*A*). Repeating this analysis in *ent1*^3−^ cells showed the basal (untreated) adenosine level reduced by 182-fold compared to WT cells (*P* = 0.002) (Fig. 2 *A* and *B*), consistent with reduced adenosine intake from extracellular media following ENT1 ablation. Furthermore, both acute and chronic treatment of decanoic in *ent1*^3−^ cells did not significantly increase adenosine levels (Fig. 2*A*). Treatment of WT cells with octanoic acid provided a nonsignificant increase of adenosine level at 1 h and 4 d, but a 237% increase after 5 d (*P* = 0.009, Fig. 2*B*). Octanoic acid treatment had no effect on adenosine levels in *ent1^3−^* cells (*P* = 0.07 for control vs. 5 d, Fig. 2*B*). Thus, both medium chain fatty acids induce an increase in adenosine levels in WT cells following chronic (5 d) treatment, and this effect was dependent upon ENT1 activity. Since this increase is only shown in the presence of ENT1 activity, and is not specific to decanoic acid, it appears unlikely that the enhanced cell proliferation shown following decanoic acid treatment in cells with reduced *ent1* expression is caused by elevated adenosine levels.

### Investigating Mechanisms Leading to Altered Decanoic Acid Response in *ent1^3−^* Cells.

To further investigate the mechanism leading to improved cell proliferation in *ent1^3−^* cells treated with decanoic acid, we initially explored altered transcriptional regulation of adenosine pathway enzymes under these conditions. In these experiments, WT and *ent1^3−^*cells were treated with decanoic acid (20 µM, 4 d), and the expression of seven genes encoding adenosine and energy-related functions (Fig. 2*C*) was assessed by qPCR. Decanoic acid treatment of WT cells did not alter expression levels of three genes involved in ß-oxidation: Acyl-CoA dehydrogenases (*ACAD8*, *ACADBS, ACOA*), and adenosine kinase (*ADK*) responsible for the first step in the use of adenosine in energy signaling by phosphorylation to adenosine monophosphate (AMP) (Fig. 2*D*). However, decanoic acid treatment caused a significant decrease in expression of adenosinedeaminase (*ADA*, *P* < 0.001) (Fig. 2*D*), responsible for conversion of adenosine into inosine, the subunit O of the ATP synthase (*ATP5O*, *P* = 0.042) responsible for ATP synthesis, ENT1 (*P* < 0.04), and AMP kinase (*AMPK*; *P* = 0.002) that functions as a global regulator of energy levels. In contrast, decanoic acid–treated *ent1*^3−^ cells showed no significant change in any of these genes (Fig. 2*E*). To directly analyze gene expression changes between the two conditions observed to enhance cell proliferation, comparison of decanoic acid treatment in WT and *ent1*^3−^ cells showed numerous changes in gene expression, with *ent1*^3−^ cells showing a relative increase in expression of all adenosine regulators (*ADA* *P* = 0.002; *ATP50* *P* = 0.004; *ADK* *P* = 0.01; *AMPK* *P* = 0.02; Fig. 2*F*), and additionally *ACADSB* (*P* = 0.02), compared to WT cells. These results highlight that decanoic acid treatment causes a significant change in expression of key adenosine-related metabolic enzymes, dependent upon ENT1 activity, and the greatest change in gene expression is found between WT and *ent1*^3−^ cells following decanoic acid treatment.

### Specificity of Decanoic Acid-Dependent Changes in Adenosine-Related Gene Expression.

**Fig. 3.**
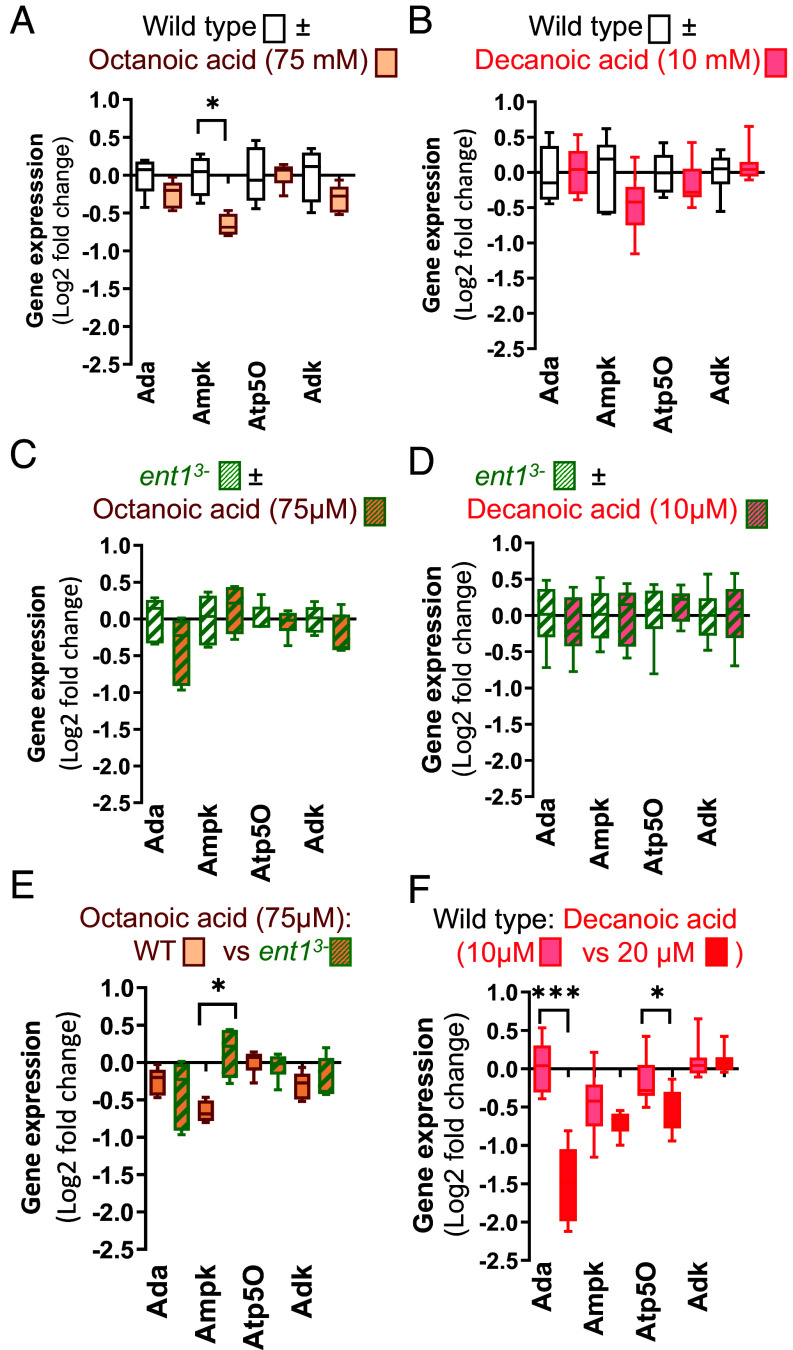
Changes in expression of key adenosine-related genes largely specific to decanoic acid at defined concentrations and dependent upon ENT1 activity. Gene expression changes of several key enzymes involved in adenosine signaling including ADA which catalyzes the irreversible deamination of adenosine to inosine, *AMPK* involved in regulating cell energy status by altered ratio of AMP/ATP, *ATP50* part of the mitochondria membrane ATP synthase involved in ATP production, and *ADK* that phosphorylates adenosine into AMP. measured by qPCR, in WT or cells under varying conditions including (*A*) in WT cells with or without octanoic acid (75 µM), (*B*) in WT cells with or without decanoic acid at reduced concentration (10 µM); (*C*) in *ent1^3-^* cells with or without octanoic acid (75 µM); (*D*) in *ent1^3-^* cells with or without decanoic acid at reduced concentration (10 µM); (*E*) in WT and *ent1^3-^* cells with octanoic acid (75 µM), (*F*) in WT cells with decanoic acid at 10 µM and 20 µM. Data are derived from three independent experiments in triplicate, with statistical analysis using a two-tailed Mann–Whitney test, *P < 0.05; **P < 0.01; ***P < 0.001.

Since initial experiments identified improved cell proliferation at defined concentrations of decanoic acid, but not for octanoic acid, we continued by investigating the specificity of fatty acids and concentration dependence for altered gene expression in WT and *ent1^3−^* cells treated for 4 d. In WT cells, we initially examined the effect of octanoic acid at 75 µM, a concentration that reduced cell proliferation to a similar level as that produced by 20 µM decanoic acid (*SI Appendix*, Fig. S7), to show no change in the expression of *ADA*, *ATP50,* and *ADK*, but a small significant decrease in *AMPK* expression following octanoic acid treatment (Fig. 3*A*, *P* = 0.02). In WT cells, reducing decanoic acid treatment from 20 µM to 10 µM blocked all changes in gene expression (Fig. 3*B*). In *ent1^3−^* cells, both octanoic acid (75 µM) and decanoic acid (10 µM) did not change gene transcription (Fig. 3 *C* and *D*). Comparison of octanoic acid treatment in WT and *ent1^3−^* cells showed a significant increase in *AMPK* expression following loss of ENT1 function (Fig. 3*E*). Comparison of decanoic acid treatment at the two concentrations, showed a significant decrease in *ADA* and *ATP50* expression at 20 µM (Fig. 3*F*, *P* < 0.001, *P* = 0.01 respectively), suggesting a dose-dependent effect on inhibiting the expression of two key proteins involved in the metabolic breakdown of adenosine to inosine and in the production of ATP. Thus, changes in the expression of adenosine signaling pathway genes and potential energy-related processes by medium chain fatty acids are dependent on the type of fatty acid and limited to a specific range of concentrations.

### The hENT1 Protein Complements Loss of *D. discoideum* ENT1 Proteins.

**Fig. 4.**
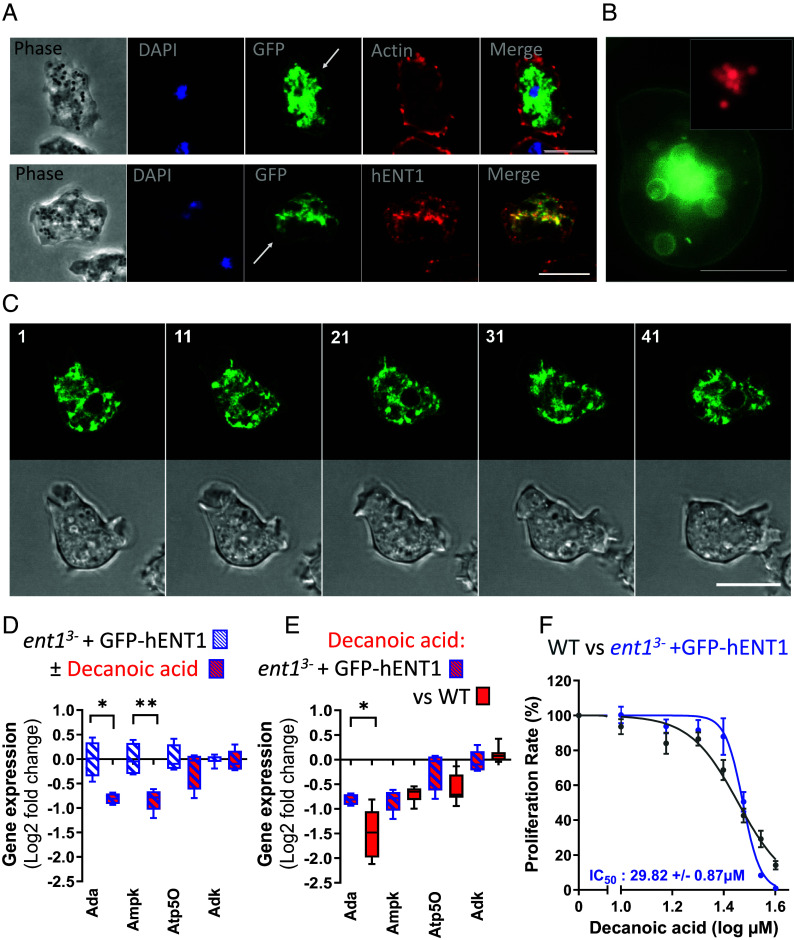
Expression of human ENT1 in the *D. discoideum ent1*^3−^ mutant restores cell and metabolic responses to decanoic acid. (*A*) Imaging fixed *ent1*^3−^:GFP-hENT1 cells by phase contrast and fluorescence microscopy (upper panel) indicates a membrane and intracellular localization of GFP-hENT1, and this localization is confirmed using a human ENT1 antibody (lower panel). (*B*) GFP-hENT1 also localizes to endocytic vesicle membranes, visualized with the uptake of Texas red-labeled dextran from media (size bar, 10 µM, also for *A*–*C*). (*C*) Time series of images of live *ent1*^3−^:GFP-hENT1 cells in fluorescence and phase contrast (time indicated in seconds). (*D*) Comparison of changes in transcription of adenosine-related genes in *ent1^3−^*:GFP-hENT1 cells, in the absence or presence of decanoic acid (20 µM), for *ADA*, *AMPK*, Atp50, and *ADK*. (*E*) Comparison of changes in transcription of adenosine-related genes following decanoic acid treatment (20 µM) between *ent1^3−^*:GFP-hENT1 and WT cells. (*F*) Secondary plots comparing proliferation rate (120-144 h) in WT and *ent1^3-^*:GFP-hENT1 cells for decanoic acid treatment. Data are derived from three independent experiments in triplicate, with statistical analysis using a two-tailed Mann–Whitney test, *P < 0.05; **P < 0.01.

To investigate the relevance of these effects to human ENT1 function, we expressed a green fluorescent protein (GFP)-tagged hENT1 protein (SLC29A1, Q99808) in *D. discoideum* WT and *ent1*^3−^ cells. The expressed protein (GFP-hENT1) was initially analyzed by Western blot using a GFP antibody to confirm the expressed protein at the predicted size of 78 kDa (*SI Appendix*, Fig. S8). Analysis of the cellular localization showed that GFP-hENT1 recruits to the plasma membrane, the contractile vacuole (CV) system, and endocytic vesicles (Fig. 4*A*), consistent with results in human cells (43). Enrichment of GFP-hENT1 on membranes of endocytic vesicles became visible by incubation of cells with fluorescent dextran uptake from the medium by macropinocytosis (Fig. 4*B* and *SI Appendix*, Fig. S9). In addition, a strong enrichment of GFP-hENT1 was detected in the CV network (45) which consists of membranous tubes and bladders and serves as an efficient osmoregulatory organelle (Fig. 4*C*). This localization is also shown in time-lapse images of live cells (*SI Appendix*, Fig. S10 and Movies S1 and S2).

The activity of GFP-hENT1 was also investigated to complement loss of *D. discoideum* ENT1 activity in *ent1*^3−^ cells. Expression of GFP-hENT1 in *ent1*^3−^ cells restored WT response to decanoic acid treatment for *ADA* (*P* = 0.028) and *AMPK* (*P* = 0.007) gene expression (Fig. 4*D*) where no significance difference was found in gene expression comparing WT and *ent1*^3−^:GFP-hENT1 cells following decanoic acid treatment (Fig. 4*E*). Finally, assessing cell proliferation response of WT and *ent1*^3−^:GFP-hENT1 cells following decanoic acid treatment (20 µM) (Fig. 4*F* and *SI Appendix*, Fig. S11), showed loss of the enhanced cell proliferation response found in the *ent1*^3−^ cells (*ent1*^3−^: GFP-hENT1, 86.6%; WT, 90.2%) demonstrating phenotypic restoration after hENT1 expression. Thus, expression of human GFP-hENT1 led to localization of the protein in cell membranes, restored gene expression changes following decanoic acid treatment, and provides similar cell proliferation rates in WT cells and the complemented mutant (*ent1*^3−^:GFP-hENT1) after decanoic acid treatment. These experiments confirm that the changes on cell proliferation and transcription observed after decanoic acid treatment are regulated by adenosine transport through ENT1 activity, and that this activity is conserved between the *D. discoideum* and human ENT1 proteins.

### ENT1 Proteins Affect Fruiting Body Size.

**Fig. 5.**
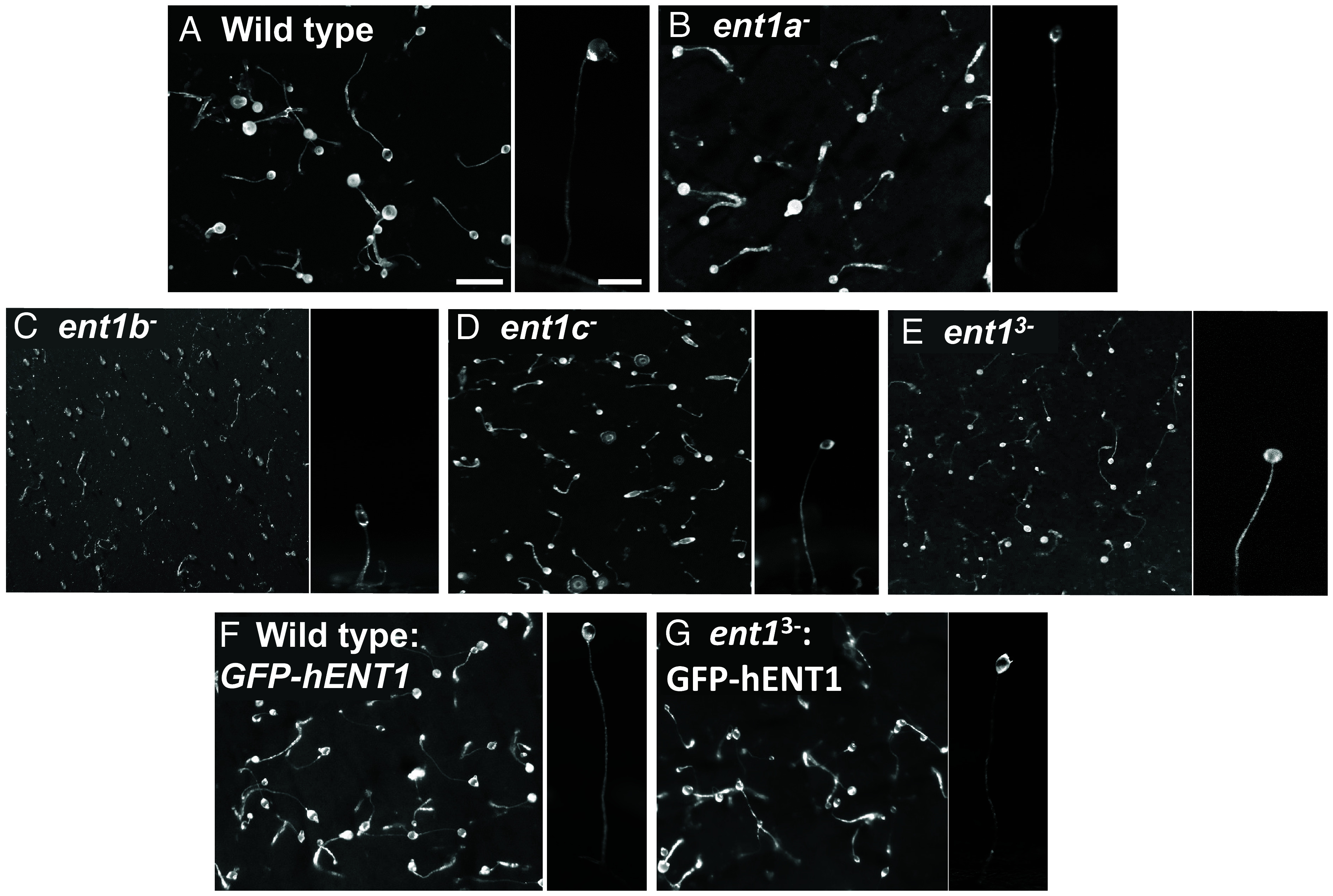
Developmental effects of ENT1 loss and rescue by the hENT1 protein. (*A*) Wild *D. discoideum* cells, starved on nitrocellulose membrane for 24 h, develop to form multicellular fruiting bodies pictured from above (*Left*), and side-on (*Right*), consisting of a round sorus held aloft by a tall think stalk. (*B*) Ablation of *ent1a* did not alter this developmental phenotype. (*C*–*E*) Ablation of either *ent1b*, or *ent1c*, or the *ent1*^3−^ in *D. discoideum* led to smaller fruiting body. Expression of the human GFP-ENT1 protein in (*F*) WT cells did not alter developmental morphology, but (*G*) partially rescued the small fruiting body size of the *ent1*^3^*^−^* mutant. Data are representative of triplicate independent experiments (*SI Appendix*, Figs. S12 and S13).

Since *D. discoideum* is also widely employed as a developmental model for the analysis of bioactive compounds (46–48), we assessed the role of ENT1 activity in multicellular development. In this model, starvation induces a series of developmental changes leading to chemotactic cell movement and multicellularity with the formation of fruiting bodies consisting of a round spore head held aloft by a thin stalk (Fig. 5), thus enabling a qualitative assessment of development following ENT1 loss or medium chain fatty acid treatment. Ablation of *ent1a* in these cells does not affect fruiting body morphology (Fig. 5), however, loss of *ent1b* or *ent1c* reduces fruiting body size. Loss of all three *ent1* genes (in *ent1^3−^*) also reduces fruiting body size (Fig. 5). Expression of GFP-hENT1 in WT cells does not alter fruiting body morphology (Fig. 5), but expression in *ent1*^3−^ cells restores fruiting body size (Fig. 5). Decanoic acid and octanoic acid treatment does not restore altered morphology in any mutant (*SI Appendix*, Figs. S12 and S13), consistent with no effect on cell proliferation following this exposure time (Fig. 1*C*).

### Decanoic Acid–Regulated Transcriptional Changes in Energy Metabolism Are Enhanced Following inhibition of ENT1.

**Fig. 6.**
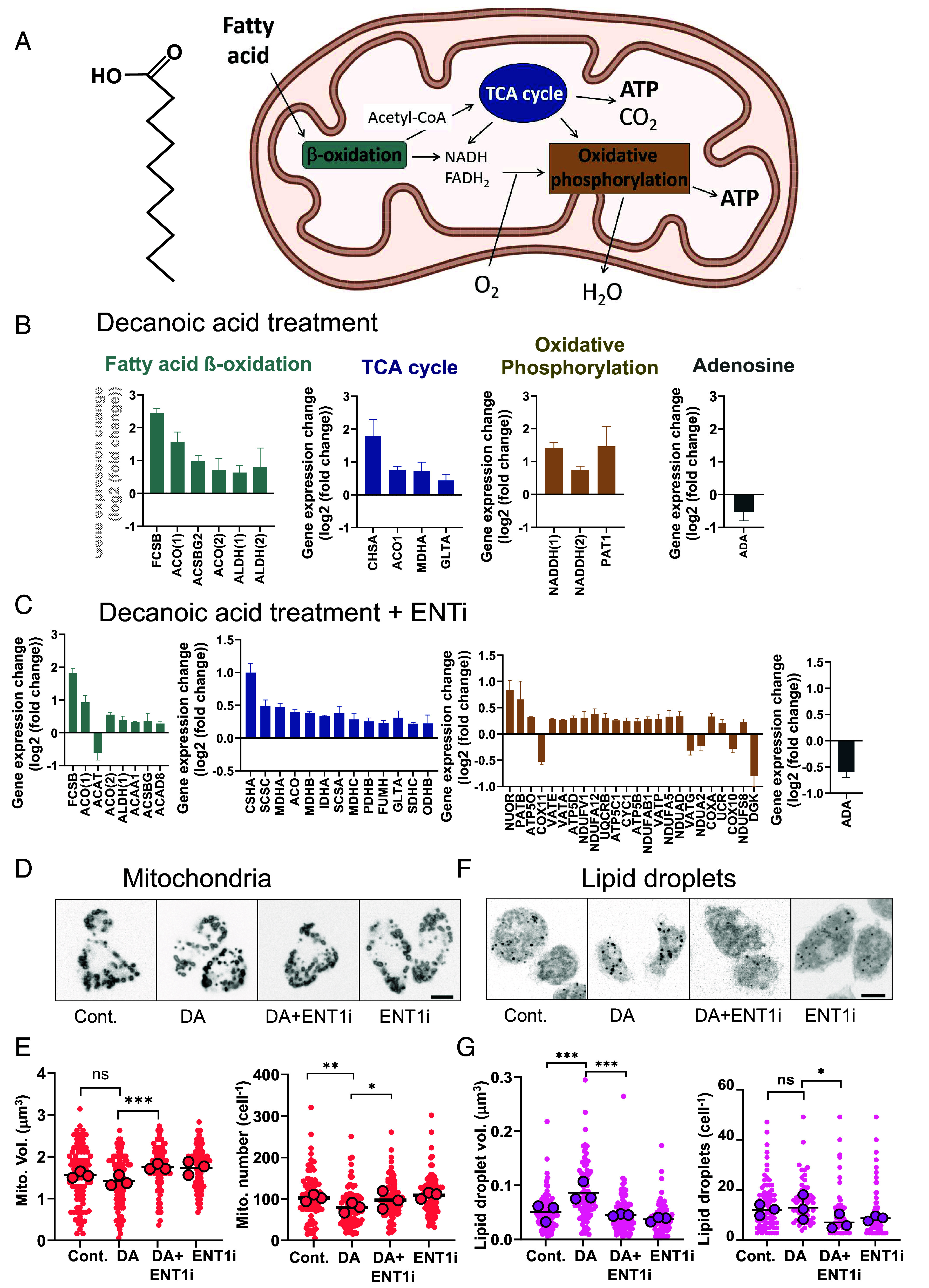
Decanoic acid treatment increases expression of genes in energy-related pathways, and pharmacological inhibition of ENT1 enhances this effect. (*A*) Mitochondrial energy regulation from fatty acid/β-oxidation provides NADH/FADH_2_ and acetyl CoA for the TCA cycle to produce ATP, and to oxidative phosphorylation to produce ATP. (*B*) In WT cells, significant (*P* < 0.05) transcriptional changes of energy metabolism-related genes were observed following decanoic acid (20 µM) 4 d treatment related to fatty acid metabolism, TCA cycle, and oxidative phosphorylation. (*C*) In WT cells treated with ENT1 inhibitor (ENT1i: NBTI, 10 µM), an increased number of significant (*P* < 0.05) transcriptional changes of energy metabolism-related genes were observed following decanoic acid (20 µM) 4 d treatment related to fatty acid metabolism, TCA cycle and oxidative phosphorylation. Data are derived from triplicate independent samples (*SI Appendix*, Tables S2 and S3). (*D*) Imaging of mitochondria with GFP-labeling in solvent-only (Cont) conditions or following decanoic acid (DA) treatment alone, or in combination with ENT1i, or with ENT1i alone, as above, and (*E*) following quantification of mitochondrial volume and number. (*F*) Imaging of lipid droplets with lipid staining following the same treatments and (*G*) quantification of lipid droplet volume and number. Data were derived from triplicate independent experiments with at least 30 cells quantified for each sample per biological repeat. Size bars (*D* and *E*) are 10 µm. Statistical analysis was one-way ANOVA with Tukeys’s multiple comparisons test. * *P* < 0.05, **P < 0.01, ***P < 0.001.

Since decanoic acid treatment induced transcriptional changes, we investigated genome-wide changes in fatty acid metabolism and energy-related gene expression (Fig. 5*A*). In these experiments, we treated WT cells with or without decanoic acid (20 µM) for 4 d and identified significant changes in comparative messenger RNA (mRNA) copy number for genes involved in fatty acid metabolism or β-oxidation, TCA cycle components, and oxidative phosphorylation following treatment (Fig. 6*A*). Decanoic acid treatment significantly increased expression of six genes in fatty acid metabolism (Fig. 6*B* and *SI Appendix*, Table S2), including a fatty acyl-CoA synthetase (*P* < 0.001) that functions as the first step in fatty acid metabolism (49) and two putative acyl-CoA synthases catalyzing the first step in fatty acid β-oxidation (ALDH1 and 2, *P* < 0.0001 for both), and a long-chain fatty acid ligase (ACSBG, *P* < 0.001). In the TCA cycle, four genes showed significantly increased expression following decanoic acid treatment, with two of these encoding potential citrate synthase orthologues (CSHA and GLTA, *P* < 0.001 and *P* < 0.05 respectively), consistent with that shown in a human neuronal cell line (SH-SY5Y) (19). Relatively few genes showed altered expression related to oxidative phosphorylation, but adenosine-related signaling confirmed a decrease in *ADA* expression (*P* = 0.04), identified in qPCR analysis (Fig. 2). These changes reflect a decanoic acid-induced increase in expression of fatty acid metabolic enzymes, and energy metabolism relating to TCA cycle and oxidative phosphorylation.

To examine a role for ENT1 activity in these decanoic acid-induced gene expression changes, we repeated this RNAseq analysis following ENT1i. In these experiments, we used a pharmacological approach to inhibit ENT1 activity (NBTI: 10 µM), to avoid long-term transcriptional changes arising from ablation of the three ENT1 proteins, in the presence or absence of decanoic acid (20 µM) for 4 d (Fig. 6*C* and *SI Appendix*, Table S3). From this analysis, expression of seven fatty acid related genes significantly increased with combinatory decanoic acid and ENT1i treatment, including four identified with decanoic acid–only treatment. Of particular importance within this group was acetyl-CoA acyltransferase (ACAA1, *P* < 0.001) encoding a protein in fatty acid peroxisomal β-oxidation, and ACAT (*P* < 0.001) encoding a protein in acetoacetyl-CoA synthesis from two acetyl-CoAs, where these effects are likely to lead to the production of acetoacetate and β-hydroxybutyrate (50). This analysis also showed an increase in ACAD8 (*P* = 0.008) expression, with this trend observed in qPCR analysis (Fig. 2*E*). Similarly, when investigating expression of enzymes in the TCA responsible for energy production in mitochondria, combined decanoic acid and ENT1i treatment significantly increased expression of 13 genes, including four that were increased with decanoic acid treatment alone, but additionally several genes encoding important TCA cycle proteins, such as two succinyl-CoA synthases (*SCSC* and *SCSA,* *P* < 0.001 for both) that couple the hydrolysis of succinyl CoA to ATP synthesis, pyruvate dehydrogenase components (*PDHB* and *ODHB,* *P* = 0.004 and *P* = 0.04 respectively) involved in the conversion of pyruvate to acetyl-CoA linking the glycolytic and TCA cycle, and fumarate hydratase (*FUMH* *P* = 0.008) responsible for converting fumarate to malate in the TCA cycle. Oxidative phosphorylation provided the greatest increase in gene expression beyond decanoic acid treatment alone, with the significant elevation of expression of 20 genes encoding electron-transport chain components including mitochondrial complex 1/2/3/4 genes, ATP synthase complex components, and proton pumps (Fig. 6*C* and *SI Appendix*, Table S3). In addition, ADA (*P* < 0.001) gene expression was also significantly reduced in these experiments. Thus, these data indicate that a reduction in ENT1 activity leads to an enhanced effect of decanoic acid in increasing transcription of genes within the TCA cycle and oxidative phosphorylation involved in energy production.

### Validating of Transcriptional Effects of Decanoic Acid Related to Energy Metabolism.

To provide functional validation of transcriptional effects in energy metabolism seen following decanoic acid treatment and in combination with pharmacological inhibition of ENT1, we analyzed both mitochondria and lipid droplets following treatment (Fig. 6 *D*–*G*), using conditions employed in the RNAseq analysis. We analyzed mitochondrial size and number, where mitochondrial volume did not change following decanoic acid treatment, but significantly increased following combination treatment with decanoic acid and ENT1i (*P* < 0.001) (Fig. 6 *D* and *E*). Similarly, combining decanoic acid and ENT1i treatment significantly increased mitochondrial number above decanoic acid–only treatment (*P* < 0.05). We also analyzed fatty acid metabolism by quantifying lipid droplet volume and number. This indicated lipid droplet volume significantly increased following decanoic acid treatment (*P* < 0.001) consistent with enhanced fat storage, and this effect was significantly reduced with the addition of ENT1i (*P* < 0.001) (Fig. 6 *F* and *G*). Lipid droplet number did not change following decanoic acid treatment but showed a significant reduction in with combined decanoic acid and ENT1i (*P* < 0.05). These data confirm that inhibition of ENT1 in the presence of decanoic acid significantly increased mitochondria volume and number, consistent with increased expression of mitochondrial genes, and combinatory treatment also decreased lipid droplet volume and number, consistent with enhanced metabolism of fats through TCA cycle and oxidative phosphorylation.

## Discussion

Pharmacological mechanisms to improve cellular energy metabolism remain largely unexplored. Since cellular energy regulation is predominantly controlled by adenosine-based signaling, it seems unusual that more research has not focused on the role of ENT1 proteins in regulating energy metabolism. This is particularly important due to the key role of medium chain fatty acids in energy-related signaling in endurance sports where elite athletes consume medium chain fatty acids for enhanced performance (2, 3), and in ketogenic diets where medium chain fatty acids boost energy at a cellular level in patients with drug-resistant epilepsy (23–26). Here, we investigate a role for medium chain fatty acid function regulated by ENT1 proteins, employing the model that was used to initially identify a direct role for decanoic acid in seizure control (32, 33, 36, 37). We show that loss of ENT1 activity leads to an unexpected increase in cell proliferation following decanoic acid treatment, but not following octanoic acid treatment. While investigating this, we show that both decanoic and less potently octanoic acid increases intracellular adenosine dependent upon ENT1 activity. However, investigating a metabolic mechanism for enhanced cell proliferation we show that decanoic acid treatment provides a significant increase in the expression of genes related to energy provision through β-oxidation, TCA cycle, and oxidative phosphorylation that is greatly strengthened following pharmacological inhibition of ENT1 activity. These results suggest a mechanism to enhance transcription of genes involved in energy provision following medium chain fatty acid exposure under low ENT1 activity conditions.

This study identified the role of ENT1 activity in *D. discoideum* to regulate cellular adenosine levels. Genetic ablation of ENT1 activity in *D. discoideum* reduced cellular adenosine levels by 182-fold, suggesting that basal levels of adenosine are primarily determined by uptake from the extracellular environment. In a similar function, human Glioblastoma-like cells show low ENT1 expression leading to elevated extracellular adenosine levels (51), thus ENT1 proteins facilitate the uptake of adenosine from extracellular matrix and are important for the regulation of cellular adenosine levels (29). In related studies, pharmacological inhibition of ENT1 with NBTI reduces seizure severity and prolongs latency in in vivo epilepsy models, likely through enhanced extracellular adenosine levels (30, 52). Thus, our study confirms an evolutionary role of ENT1 proteins in regulating intracellular adenosine levels, but additionally suggests that specific medium chain fatty acids may trigger enhanced energy conditions following inhibition of ENT1 activity.

The classical ketogenic diet involves consumption of high levels of long chain fatty acids and low levels of carbohydrate, providing a low glucose environment, and triggers increased brain adenosine levels in rodent models (16, 26), likely due to the metabolic breakdown of ATP in the synapse. To our knowledge, elevated adenosine levels have not been shown following treatment with medium chain fatty acids. Thus, surprisingly, in *D. discoideum,* decanoic (20 µM), and octanoic acid (75 µM) also trigger this increase in adenosine levels during growth in high glucose media, despite diverging from the animal lineage around a billion years ago (53). Mechanistic insight into this effect was shown by transcriptional analysis, where expression of the gene encoding the adenosine deaminase (ADA) protein that is responsible for the breakdown of adenosine to inosine (29) was reduced following decanoic acid treatment. Consistent with this outcome, ablation of this enzyme in a mouse model also increases brain adenosine levels (54). Thus, we show that medium chain fatty acid treatment elevates adenosine levels, and we provide a potential mechanism for this effect most likely through transcriptional regulation, although further translational studies in animal models are necessary to confirm this mechanism.

This study also shows several other important signaling mechanisms induced by decanoic acid treatment and relating to ketogenic diets. Treatment of WT cells significantly decreased expression of the O subunit of ATP synthase (*ATP5O*), suggesting a potential decrease in mitochondrial complex IV ATP generation, and genetic ablation of ENT1 activity reversed this effect to increase ATP50 expression. This increase was also shown in human fibroblasts following decanoic acid treatment, providing validation of this effect in human cells (55). Decanoic acid treatment also significantly reduced *ent1a* expression, potentially reducing extracellular adenosine transport. Furthermore, both decanoic acid (20 µM) and octanoic acid (75 µM), reduced *AMPK* expression. AMPK is a metabolic sensor that is activated by an increase in the ratio of cellular AMP:ATP (56, 57). Glucose reduction triggers a low energetic state, leading to AMPK activation to inhibit cell growth via reduced activity of mTORC1, and high glucose levels reverse this process, and this mechanism is highly conserved in eukaryotes (58, 59). We show that both decanoic and octanoic acid decrease expression of AMPK in WT cells, effectively triggering a high-energy environment response (56, 57).

Classical ketogenic diets have also been shown to regulate the expression of genes involved in energy production (60). The mechanisms leading to these changes were initially considered to be through reduced glucose levels triggering ketosis, leading to increased mitochondria energy production (7, 11, 26). An equivalent effect is seen with decanoic acid treatment, to enhance mitochondrial respiratory chain complex activity (19, 61), proposed through direct activation of PPARγ (20). We now extend this effect to include *D. discoideum*, where decanoic acid treatment enhances expression of mitochondrial enzymes (in β-oxidation, TCA cycle, and oxidative phosphorylation), and combinatory treatment with pharmacological inhibition of ENT1 activity widely enhances these transcriptional effects. Importantly, several genes in this study are also enhanced in the rat hippocampus with the ketogenic diet (60), including Atp5D, Atp5c1, Atp50, Cyc1, Ndufa8, and Mdh1. In both studies, expression of these genes increased by around 50%. In addition, a range NADH dehydrogenase and succinate dehydrogenase complexes proteins were also found to be up-regulated in expression in both studies. Thus, the transcriptional changes identified in our manuscript are consistent with that shown in the rat hippocampus during the ketogenic diet treatment.

Transcriptional changes in *D. discoideum* were also validated in functional studies. The enhanced expression of mitochondrial genes is likely to be through the increase in mitochondrial number and size shown following ENT1i. In addition, the enhanced expression of fatty acid metabolism genes with decanoic acid treatment, is validated through a corresponding increase in fatty acid metabolism visualize by lipid droplets reduction. This effect has been suggested in human patient-derived fibroblasts (55). Surprisingly, since *D. discoideum* lacks PPARγ orthologs, thought to be the trigger for decanoic acid-dependent mitochondrial biogenesis of PPARγ (20), data presented here suggest this decanoic acid-dependent increase in energy supply and mitochondrial load may be caused by increased fatty acid metabolism.

Interest in decanoic acid has grown over the last decade due to increasing use as a component of dietary MCT supplements for disease treatment and in exercise (35). We show that decanoic and octanoic acids provide an important role in increasing adenosine levels consistent with rodent models of a classical ketogenic diet (25). Furthermore, we show that following pharmacological reduction in ENT1 activity, decanoic acid triggers a metabolic shift to enhance expression of genes involved in TCA cycle and oxidative phosphorylation that are key energy-providing mechanisms. Importantly, these processes have been demonstrated in the presence of glucose and in the absence of ketosis, suggesting functions that are not related to ketogenesis. This work also suggests that the pharmacological inhibitors of ENT1 that are in development and reduce seizure activity (52) in combination with decanoic acid may enhance cellular energy provision.

## Materials and Methods

Detailed methods are available in *SI Appendix*.

### *D. discoideum* Growth Assay.

*D. discoideum* cells in the presence of decanoic or octanoic acid, or a dimethyl sulfoxide (DMSO) control were grown at 22 °C in HL5 medium over a 7 d period, and the cell number was quantified.

### Creation of *ent1* Null Mutants and GFP-hENT1 Construct.

*D. discoideum ent1a* and *ent1b* were ablated by insertional mutagenesis (18) and *ent1c* by CRISPR-Cas9 technology (62). hENT1 protein (SLC29A1, Q99808) was expressed in *D. discoideum* by synthesis of an encoding gene using *D. discoideum* codon bias (NBCI GenBank) and cloned into an extrachromosomal vector pDM1207 before expression in *D. discoideum*.

### Adenosine Quantification.

Cellular adenosine levels were measured by LC–MS using an Agilent 6550 iFunnel Q-ToF coupled to an Agilent 1290 infinity high-performance liquid chromatography (HPLC) (63).

### Gene Expression Analysis.

RNA was extracted from the cells (Qiagen, 74104) and cDNA synthesized (Thermo Fisher Scientific, K1622 kit). Gene expression was analyzed using qPCR and primers 75 to 150 base pairs apart within the gene (Sigma-SYBR® Green Jumpstart™ Taq ReadyMix™), with levels compared to the housekeeping gene *ACT2* (DDB_G0274133), with expression fold-change calculate using the ΔΔ-Ct method.

### Cell Imaging.

Fixed cell staining involved either phalloidin Atto 550 (Sigma-Aldrich, 19083) to visualize filamentous actin, or polyclonal rabbit anti-Ent1 antibodies (Proteintech, 29862-1-AP), followed by secondary goat anti-rabbit Alexa Fluor 555-conjugated IgG (Invitrogen, A-21428). Confocal microscopy was performed with an inverted Leica TCS SP8 (Leica Microsystems, Wetzlar, Germany).

### Cell Development Assays.

Exponentially growing cells were developed on nitrocellulose filters on absorbent pads soaked in phosphate buffer containing compound or DMSO control. Developmental phenotypes were imaged after 24 h.

### Quantification of Organelle Size and Number.

Organelles were quantified by spinning disk fluorescence microscopy. Peroxisomes were labeled using peroxisomal targeting sequence serine-lysine-leucine (SKL) fused to the C terminus of monomeric red fluorescent protein (mRFPmars); mitochondria were labeled using GFP fused to the transmembrane domain of gemA (64). Lipid droplets were stained using LipidSpot488 (Biotium) in HL5 medium.

## Supplementary Material

Appendix 01 (PDF)

Movie S1. **Time lapse imaging of *D. discoideum ent1^3-^* cells expressing GFP-hENT1**. Combined brightfield and fluorescent movies showing localisation of GFP-hENT1 (GFP-huEnt1) to plasma membranes, endosomes and contractile vacuoles. Size bar = 10 μM

Movie S2. **Time lapse imaging of *D. discoideum ent1^3-^* cells expressing GFP-hENT1**. Combined brightfield and fluorescent movies showing localisation of GFP-hENT1 460 (GFP-huEnt1) to plasma membranes, endosomes and contractile vacuoles. Size bar = 10 μM

## Data Availability

All data are included in the article and/or supporting information.
