# Supplementary material for: Pharmacological inhibition of ENT1 enhances the impact of specific dietary fats on energy metabolism gene expression
Source: Proc Natl Acad Sci U S A. 2024 Aug 29;121(36):e2321874121. doi: 10.1073/pnas.2321874121 (PMC11388398; doi:10.1073/pnas.2321874121)
Supplement: Supplementary file 1 — Appendix 01 (PDF) [file pnas.2321874121.sapp.pdf]

PNAS

Supporting Information for

Pharmacological inhibition of ENT1 enhances the impact of specific dietary fats on energy metabolism gene expression

Erwann Pain[1], Stuart Snowden[1], Joseph Oddy[2], Sonia Shinhmar[1], Yousef M.A. Alhammad[3], Jason S. King[3], Annette Müller-Taubenberger[4], and Robin SB Williams[1]*

***Corresponding Author**: Robin S.B. Williams

Email: robin.williams@rhul.ac.uk

This PDF file includes:

Supporting text

Figures S1 to S13

Tables S1 to S3

Legends for Movies S1 to S2 (Fig S10)

SI References

Other supporting materials for this manuscript include the following:

Movies S1 to S2 (Fig. S10)

Supporting Information Text

Supplementary materials and methods.

Chemicals

Decanoic and octanoic acid were purchased from Alfa Aesar (A14788, A11149 respectively). B1136.S-(4-nitrobenzyl)-6-thioinosine (NBTI/NBMPR) was purchased from Merck (N2255). Dimethyl sulphoxide (DMSO) was purchased from VWR (MFCD00002089).

***D. discoideum* cell culture**

D. discoideum cells were cultivated in 60-mm Petri dishes in axenic media (HL5 medium, Formedium), at 22 °C under/with constant light.

***D. discoideum* cell proliferation assay**

Cell proliferation was measured in liquid culture (HL5 media, Formedium), at 22 °C in 24-well plates, and treated with varying concentrations of decanoic or octanoic acid (A14788 and A11149, Alfa Aesar) at a constant solvent level (DMSO, 0.2%). Cells were incubated for seven days, and cell densities were determined from day 3 to day 7 and normalised to the solvent-only control.

Genetic manipulation

D. discoideum ent1a and *ent1b* genes were ablation using homologous recombination. For each gene, knockout cassettes were generated by PCR amplification of the 5' and 3' regions, creating homologous regions that were used to recombine with the target gene with primers including restriction enzyme sites (NcoI and KpnI or Ngo MIV and Hind III in the 5' arm of the *ent1a* and *ent1b* genes respectively (5' and 3' of the arm respectively) and SpeI and PstI or Bgl II and Spe I cut sites were inserted into the 3' arm of the *ent1a* and ent1b genes (5' and 3' of the arm respectively) (Table S1). These two regions were sequentially cloned into the pLPBLP vector at sites flanking the blasticidin-resistance gene for ent1a knock out, and pDM1081 (Addgene, 108981) at site flanking the hygromycin-resistance gene for the ent1b knock out. Knockout constructs were transfected into *D. discoideum* resulting in a disruption to the ent1a and b genes via homologous recombination, which was verified by PCR amplification of genomic control, vector control and knockout bands (Supplementary Fig. 3). Homologous recombination resulted in ablation of ent1a and b expression, RT-PCR was carried out. RNA was extracted from wild type and knockout cells using RNeasy mini kit (according to manufacturer's protocol). To avoid DNA contamination in RNA extracts the DNA-free kit (according to manufacturer's protocol). cDNA was synthesized using the RevertAid First Strand cDNA synthesis kit using oligo(dT)18 primers (according to manufacturer's protocol). PCR amplification was carried out of the knockout gene and Ig7 (positive control housekeeping gene, which produces the mitochondrial large subunit rRNA (DDB_G0294034) (1).

D. discoideum ent1c was ablated using CRISPR-Cas9 technology. Here, plasmid pTM1285 (NBRP Nenkin, G90426) was used to generate the knock-out vector. Three guides composed of two oligonucleotides (forward and reverse) were selected within *ent1c* gene (Dictybase, DDB_G0281513) using Crispor (http://crispor.tefor.net/) to identify Protospacer Adjacent Motifs (PAMs), and restriction enzyme site for *Bpi* I (Thermo Fisher, ER1012) were added on each guide using PCR. Oligonucleotides of each guide were phosphorylated and annealed using 10 µM of each oligonucleotide, 1 µl of 10X T4 Ligation buffer (New England Biolabs, B0202), 0.5 µl T4 Polynucleotide kinase (10 U/µl, New England Biolads, M0201), and completed with molecular grade ddH$_2$O (Thermo Fisher, J71786.K2) for a total reaction volume of 10 µl using a thermocycler (37 °C 30 min, 95 °C 5 min, ramp down to 25 °C at 5°C/min).Each obtained annealed oligonucleotides was digested and ligated to pTM1285 using restriction enzyme *Bpi* I (Thermo Fisher, ER1012), 2 µl 10X Tango buffer (Thermo Fisher, BY5), 1 mM dithiothreitol (Merck, DTT-RO), 1 mM ATP (New England Biolabs, P0756S), 1 µl T4 DNA ligase (Thermo Fisher, EL0011). Reaction volume was adjusted to 20 µl using molecular grade ddH$_2$O and digestion/ligation was carried out on a thermocycler (37 °C 5 min, 23 °C 5 min, 6 cycles). The obtained knock-out vector was transfected in competent cells (*E. coli*) by heat shock at 42 °C for

1 min and grown overnight in LB agar with ampicillin. Resistant colonies were put to grow overnight in liquid LB with ampicillin, and the resistance vector was extracted with the Plasmid Plus Maxi Kit (Qiagen, 27104). *D. discoideum* cells were transfected with 3µg/plasmid containing each guide by electroporation. Cells were left to recover in HL5 medium for 8h and selected using 10µg/ml neomycin (Merck, A1720) for 72 h. Genomic DNA of resistant colonies was extracted using the Wizard Genomic DNA Purification kit (Promega, A1120), and potentially mutated sequences were amplified with a set of primers designed before and after the three CRISPR guides in the *ENT1C* using PCR (CRISPR ENT Screen primers Table S1). Amplified sequences were sent for sanger sequencing and disruption of the open reading frame in resistant mutants was found by comparing wild type and mutant sequences using Synthego ICE (https://ice.synthego.com/#/) (Supplementary Fig. 4).

Quantification of adenosine level by liquid chromatography mass spectrometry.

Wild type (Ax3) or *ent1*$^{3-}$ mutant cells were treated for 1 h, 4 or 5 days with decanoic or octanoic acid, and 10^7 cells were used for sample analysis. Extraction of aqueous phase was achieved by adding HPLC grade tert-butyl methyl ether (1634-04-4, Thermo Scientific) and 80/20 methanol:water containing 200 nM L-valine (72-18-4, Merck) to the samples before incubation at 4 °C for 10 min. HPLC grade water (7732-18-5, Thermo scientific) containing 0.15 mM ammonium formate (540-69-2, Merck) was added, and aqueous phase was transferred in glass vials after centrifugation. Sample analysis was carried out using an Agilent 6550 iFunnel Q-ToF coupled to an Agilent 1290 infinity HPLC (2).

Quantification of gene expression by qPCR

Cells were seeded at $2x10^4$ cells/ml in 60-mm Petri dishes and treated for 4 days with 20 µM decanoic acid, 75 µM octanoic acid or with solvent only (DMSO 0.2%) as the control. Following treatment, RNA was extracted from the cells (Qiagen, 74104) and cDNA synthesised (Thermo Fisher Scientific, K1622 kit). Gene expression was analysed using qPCR and primers 75-150 base pairs apart within the gene (Sigma-SYBR® Green Jumpstart™ Taq ReadyMix™), with levels compared to the housekeeping gene *ACT2* (DDB_G0274133), with expression fold-change calculate using the ΔΔ-Ct method (Table S1).

Quantification of protein expression by Western blotting

Protein samples were prepared by directly lysing 7.5 x 10^7 cells/ml cells in 2 x Laemmli buffer (0.004% bromophenol blue, 10% 2-mercaptoethanol, 20% glycerol, 4% SDS, 0.125 M Tris-HCl) followed by boiling at 96 °C for 6.5 min. Protein samples were prepared using RIPA buffer and protease cocktail inhibitor and stored in 6X loading buffer and boiled for 5 min at 100 °C. 6 - 20 µl of each sample was separated by sodium dodecyl sulphate polyacrylamide gel electrophoresis (stacking gel: 5%, resolving gel: 10 - 15%). After proteins were transferred to a polyvinylidene difluoride membrane (pore size 0.45 µm Millipore, Amersham), the membrane was stained with Ponceau S dye and then blocked for 1h with blocking buffer (LI-COR). The membrane was incubated overnight at 4 °C with primary antibodies detecting GFP (1:1000, 3H9, Chromotek). The primary antibody was dissolved in blocking buffer (LI-COR) containing 0.1% Tween 20. As a loading control, streptavidin Alexa Fluor 680 conjugate (1:5000, Invitrogen, S21378) was used. The membrane was washed with TBST and subsequently incubated for 1 h with DyLight800 goat anti-rat IgG (1:10000, Thermo Fisher) diluted in blocking buffer containing 0.1% Tween 20. After the membrane was washed with TBST, protein levels were visualised using the Odyssey CLx imaging system (LI-COR).

Over-expression of human ENT1

Human ENT1 protein (SLC29A1, Q99808) was expressed in *Dictyostelium discoideum* by synthesis of an encoding gene using *D. discoideum* codon bias (NBCI GenBank), with a *Bgl* II cut site added to the beginning of the sequence, and a *Spe* I cut site after the stop codon at the end of the sequence. The resulting *hENT1* DNA sequence was purchased from Twist Bioscience

(https://www.twistbioscience.com/). The plasmid pDM1207 (Addgene, 108984), an N-terminal GFP plasmid using the promoter of coactosin (*COAA*, DDB_G0293898), an actin binding protein, was used as the extrachromosomal vector. hENT1 and pDM1207 were cut using *Spe* I and *Bgl* II (Thermo Fisher, ER1252 and ER0082) for 1 h at 37 °C, ligated using T4 DNA ligase (Thermo Fisher, EL0011) for 1.5 h at room temperature, and transformed into competent bacteria (*E. coli TOP10*) (heat shock at 42 °C for 1 min) to be grown overnight on LB agar (Merck, L7025) with ampicillin (Merck, A9518). Resistant colonies were put to grow overnight on liquid LB with ampicillin (Merck, A9518), and DNA of liquid grow bacteria was extracted with the GeneJET Plasmid Miniprep kit (Thermo Fisher, K0502). Correct insertion of the *hENT1* DNA sequence in pDM1207 was assessed by sanger sequencing on the extrachromosomal vector using primers at the beginning and the end of *hENT1* gene (hENT1 5' and 3' seq primers, Table S1).

The *hENT1* containing extrachromosomal vector was electroporated into *D. discoideum* cells. Transformed overexpressor cells were selected and maintained with 10 µg/ml neomycin (Merck, A1720).

Fluorescence and live-cell microscopy

First, cells expressing GFP-hENT1 were assessed using standard fluorescence microscopy. Cells were washed and resuspended in KK2 or LoFlo medium (Formedium, LF0501) containing 0.5 mg/ml Texas Red dextran (MW 10,000; Thermo Fisher, D1828), and incubated for 1 h in the dark before being photographed using an Olympus IX71 wide-field fluorescence microscope at 96 x magnification. Images were captured using a QICAM FAST 1394 camera.

For immunolabeling, *D. discoideum* Ent1 triple-null cells expressing GFP-hEnt1, were seeded on coverslips, fixed with picric acid/paraformaldehyde for 20 minutes, and then post-fixed with 70% ethanol for 10 min. After blocking, the cells were washed three times with PBS and stained with either phalloidin Atto 550 (Sigma-Aldrich, 19083) to visualise filamentous actin, or incubated with polyclonal rabbit anti-Ent1 antibodies (Proteintech, 29862-1-AP), followed by secondary goat anti-rabbit Alexa Fluor 555-conjugated IgG (Invitrogen, A-21428). DNA was visualised by staining with DAPI. After immunostaining, samples were washed three times in PBS and embedded using Dako mounting medium (Agilent Technologies).

Confocal microscopy was performed with an inverted Leica TCS SP8 (Leica Microsystems, Wetzlar, Germany) equipped with lasers for 405, 488, 552, and 638 nm excitation. Images were acquired with a HC PL APO 63x/1.40 oil PH3 objective. Recording was sequential to avoid bleed-through. GFP, Atto-550 and Alexa-Fluor 555 were recorded with the hybrid photo detectors, DAPI with the conventional photomultiplier tube.

For live-cell microscopy, cells were seeded in µ-dishes (Ibidi, Germany), incubated in LoFlo medium for about 1 h, and overlaid with a thin agarose sheet before imaging. Confocal microscopy of live cells was performed using an inverted Zeiss LSM 780 confocal microscope equipped with a Plan-Apo 63x/NA 1.46 oil immersion objective (Zeiss AG, Oberkochen, Germany). For image analysis, LAS X (Leica) or ZEN (Zeiss) software was used, and images were processed using ImageJ/Fiji software (http://imagej.nih.gov/ij) applying linear enhancement for brightness and contrast.

Development assays

D. discoideum cells (1 x10^7) were centrifuged at 500 *g* for 3 min, washed twice by resuspension in KK2 buffer (16.2 mM KH_2PO_4, 4 mM K_2HPO_4), and resuspended in 1 ml of KK2 buffer, and placed on nitrocellulose filter(3). Decanoic and octanoic acid and, at indicated final concentrations (500 µl) was added to absorbent pads and placed beneath nitrocellulose filters. Cells were incubated in a humid environment for 20-22 h at 22 °C. Fruiting body morphology was recorded using a dissection microscope with a camera and Qcapture software.

Quantification of gene expression by qPCR

D. discoideum cells were seeded at 2 x10^4 cells/ml in 100 mm culture dishes and treated for 4 days with 20 µM decanoic acid or 75 µM octanoic acid. Medium was changed every 48 h. Following treatment, mRNA was extracted from the cells using an RNeasy kit (Qiagen, 74104), potential DNA contamination was removed using a DNA removal kit (Thermo Fisher, AM1906) and cDNA was synthesised using RevertAid cDNA Synthesis kit (Thermo Fisher, K1622). Primers (Table S1) 75-200 base pairs apart within the genes were selected for amplification with a qPCR readyMix (Merck, SYBR® Green Jumpstart™ Taq ReadyMix™) and gene expression was analysed using qPCR (Rotor Gene 6000, Qiagen). Expression levels were compared to the housekeeping gene *ACT2* (DDB_G0274133), and the expression fold-change was calculated using the ΔΔ-Ct method. Change in expression was showed as log(2) of fold-change.

Table S1 Primers

Primer name	Primer sequence
1	ATAGGGAAATGAAGATGATAAAG
2	ATAGCCGGCCAACCAATGGTAAATAAAATG
3	ATAAAGCTTGATAAACAACCAATTGAAAGC
4	CGATCAGAAACTTCTCGACAGACGTCGCGGTGAG
5	CGATAGTGGAAACCGACGCCCC
6	ATAAGATCTGGTTCACTTGTATTTTTTTGTAC
7	ATAACTAGTCATGATAATACCAGATAACTCTC
8	ATACTGAACCTAATAATAAACCAATG
ENT1B RNA 5' Rev	ATAGTTGATGCAATTTTATATTTCTCTGG
ENT1B RNA 3' Fwd	ATAGGTGTTATAGTTGTAATAACAAG
Ig7 Fwd	GTACTGTGAAGGAAAGATGAAAAG
Ig7 Rev	CTATGGACCTTAGCGCTC
Guide 1	Fwd : AGCATTTGTTGATGGCTTATCTCA<br>Rev : AAACTGAGATAAGCCATCAACAAA
Guide 2	Fwd : AAACAGATACTCAACAATAGCTGT<br>Rev AGCAACAGCTATTGTTGAGTATCT
Guide 3	Fwd AGCATACTCAACAATAGCTGTAAC<br>Rev AAACGTTACAGCTATTGTTGAGTA
CRISPR *ENT1* Screen	Fwd CTACATTTCCATGTATTTATATGG<br>Rev 5' CTATTATTGTCTGATGAAAATG
CRISPR *CBFB* Screen	Fwd GGTACGATAGCTTTCAATCAAG<br>Rev CTACCATTGTTATTAGTATTTGG
hENT1 5' seq	GGTAATAATGTACCTAAACCTAAC
hENT1 3' seq	GCTTTAGGTGCTGTTTTTAG
ACT2 (DDB_G0274133)	Fwd 5' CGGTTCTGGTATGTGTAAAGC 3'<br>Rev 5' CCTCTTTTTGATTGAGCTTCATC 3'
ACAD8 (DDB_G0288647)	Fwd 5' CGTCGTACATTCTTTAATATTG 3'<br>Rev 5' CTTCTCTCATCACATCTCTTG 3'
ACADSB (DDB_G0282967)	Fwd 5' CCAATTACAACATTATCAGAAGAG 3'<br>Rev 5' CCCATAAAATTCATATTGGCTCC 3'
ACOA (DDB_G0283261)	Fwd 5' GGATAAAGCAACATTAAGAAGAATAG 3'<br>Rev 5' CTATCACGATCAAAATGAAATTCAC 3'
ADA (DDB_G0287371)	Fwd 5' CCTATGATCAAAATGAATTGGC 3'<br>Rev 5' CTAAATAGCTAACTCCATCTGC 3'
ADK (DDB_G0286057)	Fwd 5' CCATTACTCGACCTTTCAACAC 3'<br>Rev 5' CATCCATTGACAAACACGTG 3'
AMPK (DDB_G0272542)	Fwd 5' GGGGATGAAGATGAAGAATTC 3'<br>Rev 5' CCACCACCAACTCCTTCAG 3'
ATP50 (DDB_G0283283)	Fwd 5' GTTAGCTCGTTTCTCAAGAAC 3'<br>Rev 5' GACTGCATCTTTGAATTGTGG 3'
ENT1 (DDB_G0283439)	Fwd 5' GAAACATCAAAAGCACCATTAG 3'<br>Rev 5' CTGATGCTGCTACATAACAATTG 3'

Quantification of adenosine level by Liquid Chromatography Mass Spectrometry

Cell treatment

Wild type (Ax3) or *ent1*$^{3-}$ cells were treated for 1h, 4 or 5 days with 20 µM decanoic or 75 µM octanoic acid, and media was changed every 48 h. 10^7 cells were collected, centrifuged at 500 g for 3 min, washed with KK2 buffer twice and used for samples analysis.

Western analysis

As a loading control, using streptavidin Alexa Fluor 680 conjugate (1:5000, Invitrogen, S21378) to bind methylcrotonyl-CoA carboxylase (MCCC1)(4).

RNA sequencing

D. Discoideum were seeded at 2.10^4 cells/ml in 100 mm culture dishes. Cells were treated with either 20 µM decanoic acid, 75 µM octanoic acid, 10 µM NBTI or 10 µM NBTI+20 µM decanoic acid for four days. Total RNA from samples was extracted using the RNeasy kit (Qiagen, 74104) and samples were diluted to 20ng/µl to be analysed by sequencing.

Sequencing of mRNA from total RNA and pathway enrichment analysis was carried out by Novogene. mRNA was purified from total RNA using magnetic bead, and the obtained samples were fragmented in 50 bp mRNA fragments. cDNA was synthesized from mRNA fragments ligated with adapters, amplified using real-time PCR and sequenced on an Illumina platform. Raw reads were cleaned by removing reads containing adapter, read containing poly-N and low-quality reads using fastp software. Reads were aligned to a known genome using Hisat2, and quantification of genes expression was carried out using FeatureCounts. Differential gene expression was assessed by using the DESeq2 R package, and differential pathway analysis was carried out using Gene Set Enrichment Analysis (GSEA, http://www.broadinstitute.org/gsea/index.jsp) with the KEGG database (https://www.genome.jp/kegg/).

Quantification of organelle size and number

Organelles were observed and quantified by spinning disk fluorescence microscopy. Peroxisomes were labelled using plasmid pJSK391, which expresses the peroxisomal targeting sequence SKL fused to the C-terminus of mRFPmars; mitochondria were labelled using pJSK528 which expresses GFP fused to the transmembrane domain of gemA (4). Lipid droplets were stained using LipidSpot488 (Biotium) diluted 1:1000 in HL5 medium.

Cells were treated as indicated, before seeding in filtered HL5 medium (with appropriate treatments) in glass bottom microscopy dishes (Mat-Tek P35G-1.5-14-C) and imaging live on a Nikon W1 Spinning Disk confocal with a Plan Apo λ 100×1.4NA oil objective using a Prime 95B A19F203018 camera. A full z-stack of cells was captured in 0.2µm increments. At least 3 fields of view were taken per sample and the experiment repeated on 3 independent days.

Organelle number and volume were then obtained using an automated analysis pipeline on the Arivis Vision 4Dx64 software optimized for each reporter. This was based on the BlobFinder tool with variables manually adjusted and until accurate segmentation could be achieved and visually verified. At least 30 cells were quantified for each sample per biological repeat.

Statistics: Data are represented as mean ± SD. Statistical significance between two groups was analysed using a two-tailed Mann-Whitney test (GraphPad PRISM).

Supplementary Figures

```
tr|Q54TT2|Q54TT2_DICDI      MGNEDDKELLEIQPMVNKMKSSSEFTNDSTDGAVGNNNNNNNKTMCNFGID---EDYEI-    56
tr|Q54TT3|Q54TT3_DICDI      MENNFIDTVIGENIITDSINST----SNSLKSSVS-----STETTCNKSIINNEENYNDD    51
sp|Q99808|S29A1_HUMAN       ------------------------------------------------------------    0
tr|Q54R17|Q54R17_DICDI      ------------------------------------------------------------    0

tr|Q54TT2|Q54TT2_DICDI      ---PIDSSIDSSNTTAFDNNNNNNSFIYTSKELEL--LKNKVDPKIAFCMTMLSIGCLSP    111
tr|Q54TT3|Q54TT3_DICDI      QKKEILEIINLSDDDEISSDTIKTEFEIED-INKN--QIKKIDKNIAFLIFILGMGNIIP    108
sp|Q99808|S29A1_HUMAN       --------------------------------MTTSHQPQDRYKAVWLIFFMLGLGTLLP    28
tr|Q54R17|Q54R17_DICDI      ----------MT---------NRDEYSPLVDITETSKAPLDKNGLAWICFLILGVGLLFP    41
                                                                    .      : : :*.:* : *

tr|Q54TT2|Q54TT2_DICDI      FHCYLASLDYFNIIYPEKYKIASTFP-------------------------FIYMTMITI    146
tr|Q54TT3|Q54TT3_DICDI      FQTFLASLDYLDNIFP-QYKMASTFP-------------------------CIYMVVICV    142
sp|Q99808|S29A1_HUMAN       WNFFMTATQYFTNRLDMSQNVSLVTAELSKDAQASAAPAAPLPERNSLSAIFNNVMTLCA    88
tr|Q54R17|Q54R17_DICDI      FNCYVAASDYFSDLYGDSYSFLMSLA-------------------------YNYIQ----    72
                            :: :::: :*:      . ..                                 :

tr|Q54TT2|Q54TT2_DICDI      TF-VILI---------KYSDKLKHHIIILSGFSFYVIVL-IIIPCLNLSKIGGSLTSYIL    195
tr|Q54TT3|Q54TT3_DICDI      TF-IVLL---------RFQNKFKSHIILSIGFPCYIVLM-ILTPIVTIVS-HTPITTYLV    190
sp|Q99808|S29A1_HUMAN       MLPLLLFTYLNSFLHQRIPQSVR-----ILGSLVAILLVFLITAILVKVQ-LDALPFFVI    142
tr|Q54R17|Q54R17_DICDI      ----WLLLFVSIFVMPRFSFKSRTILFLLAGSLILFYMPFN-NMIFGR----NEKVSMGI    123
                                 *:         :   . :       *    . :       .             :

tr|Q54TT2|Q54TT2_DICDI      TLLFIAITAIFDGMIQGSVFALASLFGSQYLLFCQIGIGLAGVIVVITRLICKLSFSNTI    255
tr|Q54TT3|Q54TT3_DICDI      ILLLMALCSFVDGLSQGTIYAYASKFGPRYSTIAVIGNGVAGVFVVLTRLICKLSFSSDN    250
sp|Q99808|S29A1_HUMAN       TMIKIVLINSFGAILQGSLFGLAGLLPASYTAPIMSGQGLAGFFASVAMICA----IASG    198
tr|Q54R17|Q54R17_DICDI      SLLCTFASGCLASLLFGTVLGLVALFPGEYTGAVMSGNGVAGMIAMALQIITKVSVPATA    183
                             ::       . .:   *:: . .. :   *      * *:**.:.    :

tr|Q54TT2|Q54TT2_DICDI      NDKVSLKIGSLVFFCTSSFLVICTLITFILILKLPIGDIIKKKKTNQDYNENPITLDGNN    315
tr|Q54TT3|Q54TT3_DICDI      N---SKKIGLIVYFIISAIIILIAITTFFYSLKIERIR--K------------ILITNNN    293
sp|Q99808|S29A1_HUMAN       S---ELSESAFGYFITACAVIILTIICYLGLPRLEFYRYYQQLKLEGPGEQET-KLDLIS    254
tr|Q54R17|Q54R17_DICDI      H---GNQESGLIFFFLAGGVLIICLLCFLVLLQLPITKYYLANFEASKLKENG-SVNGIE    239
                                  . . : :*  :  :::  :  ::   ::                     :   .

tr|Q54TT2|Q54TT2_DICDI      NNDNNNNNNNNENNNNENNNNNNNINIEIDNFEEIYSPFKFTFKKNLKYSAMLSFLFTMT    375
tr|Q54TT3|Q54TT3_DICDI      NN---NNKNQ-----IENDNQVNNIN------GKEKHPFKEVFKKTYGFGFMVFYNFVIV    339
sp|Q99808|S29A1_HUMAN       KGEEPRA-----------GKEESGVSVSNSQPTNESHSIKAILKNISVLAFSVCFIFTIT    303
tr|Q54R17|Q54R17_DICDI      SGDGDAK-----------PKK------SAR---QWMGELLNILKKVWREALVVFTVFFTT    279
                            ..                 ::            :    :   :*:    .  :   *  .

tr|Q54TT2|Q54TT2_DICDI      LFVFPGIVIQIKS-D--RIERSW----WIFSLIAVYNIADSLGKALPLIVHKNDKRIPSV    428
tr|Q54TT3|Q54TT3_DICDI      LFLFPGIVVRIESLH--GIKSDW----WVFIIIAVYNTSDCIGKTLFSIFN---YIILPL    390
sp|Q99808|S29A1_HUMAN       IGMFPAVTVEVKSSIAG--SSTWERYFIPVSCFLTFNIFDWLGRSLTAVFM---WPGKDS    358
tr|Q54R17|Q54R17_DICDI      LSIFPGLTQLIQTSNEHQLSSDW----FIIVFFSIFMVGDFIGRTVPKWFI---IFTPSN    332
                            : :**.:.  :::      .  *      .  :  :   * :*:::   .

tr|Q54TT2|Q54TT2_DICDI      PWLWFISIGRCIFIVFFIIAN-----YYSNIFTHESLIYLFLFIFAFSNGYISSIALSQS    483
tr|Q54TT3|Q54TT3_DICDI      KLVWVVLIGKSIFVLLFFLCI------YNDNFNHEQMVIIFLIIFGVLSGGVVSYGVSEG    444
sp|Q99808|S29A1_HUMAN       RWLPSLVLARLVFVPLLLLCNIKPRRYLTVVFEHDAWFIFFMAAFAFSNGYLASLCMCFG    418
tr|Q54R17|Q54R17_DICDI      LWIPTFL--RLAFFPLFALCI------KPLVFNNNAWYFVFMFIFSISNGYCGTLAMIFG    384
                              :  .   :  *. :: :.           * ::    .*:  *.. .*   :  :  .

tr|Q54TT2|Q54TT2_DICDI      PSTVPPKYRELSGIIMSSALNIGLLLGSVFNLIFVFAQK-------          522
tr|Q54TT3|Q54TT3_DICDI      PKRVEEKYKPSCSVFLSLALNIGLMSGSSLNLLVSFFM--------          482
sp|Q99808|S29A1_HUMAN       PKKVKPAEAETAGAIMAFFLCLGLALGAVFSFLFRAIV--------          456
tr|Q54R17|Q54R17_DICDI      PTKAEEHEKEYAGIIMSFFLNFGIWVSTHFAFLLSYLVTGSTGINF          430
                            *. .       .. :::  * :*:  .: : ::.
```

Fig. S1. Alignment of *Dictyostelium* and human ENT1 protein identifying conserved amino acids. Conserved catalytic amino acids (in the human protein) highlighted in blue. Uniprot identifiers given. Amino acid position is labelled. * = conserved residues, : = indicates conservation between groups of strongly similar properties, .= conservation between groups of weakly similar properties. Boxed regions represent catalytic amino acids.

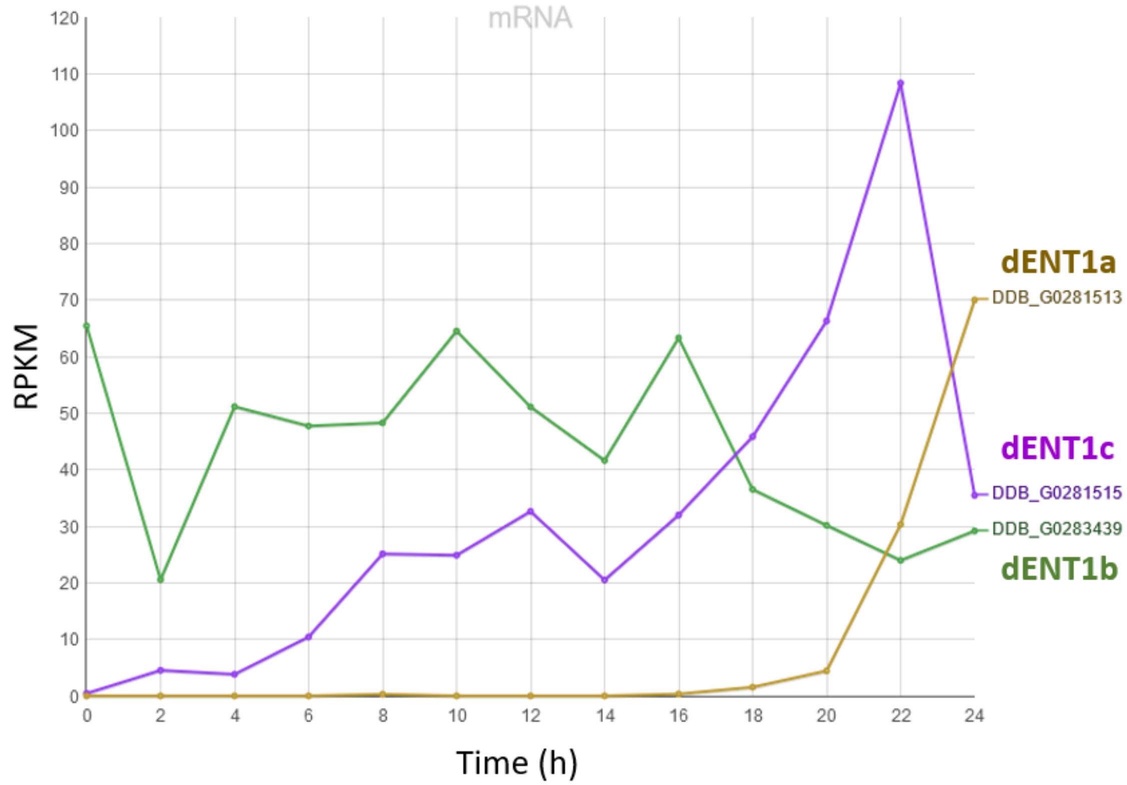

Fig. S2. Transcriptional analysis of *Dictyostelium* ENT1 orthologues during development. Comparative expression data for dENT1a, DDB_G0281513, Uniprot Q54TT3 (482 aa); dENT1b DDB_G0283439, Uniprot Q54R17 (430 aa); and dENT1c, DDB_G0281515, Uniprot Q54TT2 (522 aa) using RNAseq analysis (5).

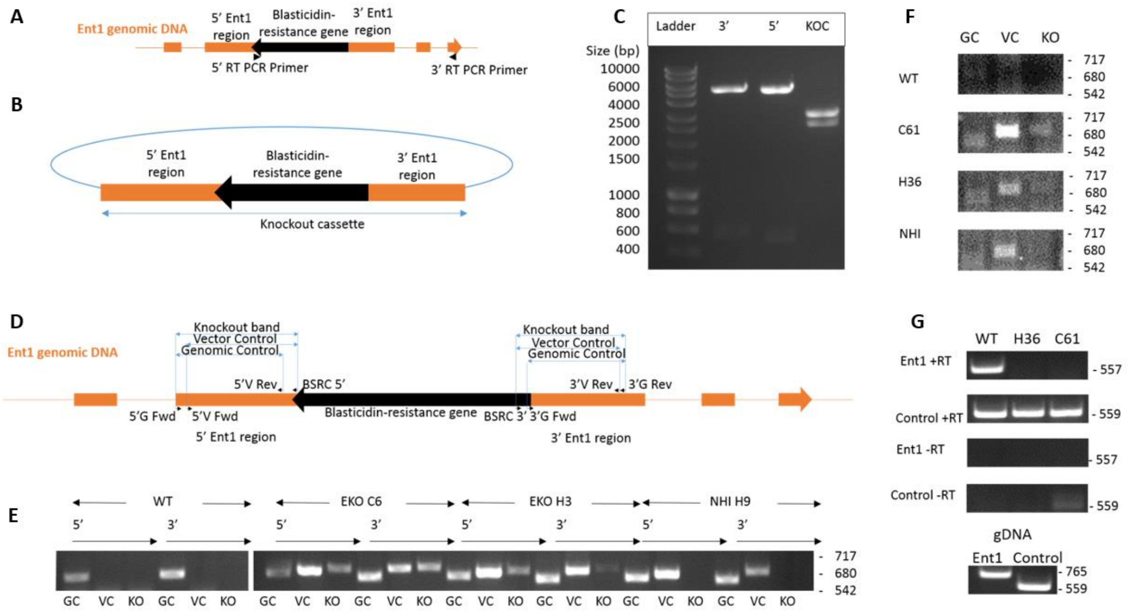

Fig. S3. Generation of an Ent1a knockout-construct. A: A diagram of Ent1a, the 5' and 3' regions are used to create the knockout construct. B: The Ent1a knockout plasmid containing the 5' and 3' Ent1a regions flanking the blasticidin-resistance gene. C: To create an Ent1a knockout *D. discoideum* a blasticidin-resistance gene was inserted into the *ent1a* gene to disrupt expression. Homologous recombination was used to insert the blasticidin-resistance gene. A plasmid had 3' and 5' regions of the *ent1a* gene inserted so they were flanking the blasticidin-resistance gene. This image shows two plasmids, with both the 3' and 5' fragments inserted. D: A blasticidin-resistance gene was inserted into the *ent1a* gene. PCR was used to confirm this, using the primers labelled on the gene. E: DNA from Ent1a knockout screen cells was isolated and PCR amplification was used to detect the presence of the blasticidin-resistance gene inserted into the correct region of Ent1a to knockout the gene. The wildtype cell line (WT) had only genomic control (GC) bands. The two wildtype cell lines that had successful insertions of the blasticidin-resistance gene into the Ent1a gene (EKO) have genomic control bands, vector control (VC) bands and knockout bands (KO). The unsuccessful insertion of blasticidin-resistance gene into the Ent1a gene (NHI) had only genomic control and vector control bands. F: Two independent isogenic cell lines with insertions were re-analysed by PCR to confirm loss of the deleted region. PCR amplification from wildtype (WT) DNA showed a product for control genomic primers, in contrast, two isogenic cell lines showed a product all three bands; both the 5' and 3' ends of the Ent1a KO construct. In the other isogenic cell lines non-homologous integrants (NIH) were found, where not all three bands were present, therefore the knockout was not successful in these cells G: Reverse transcriptase PCR of WT and Ent1 KO strains (H36 and C61). A PCR band of expected size (557 bp) is only shown in cDNA derived from WT cells (Ent1a + RT), although cDNA from both WT and the two mutants contained control (Ig7) cDNA. No DNA contamination was present, since samples without reverse transcriptase (-RT) did not show amplified products. The primer sites are shown in A, however, in the mutants the primer will not bind as it is in the region deleted by the blasticidin-resistance cassette.

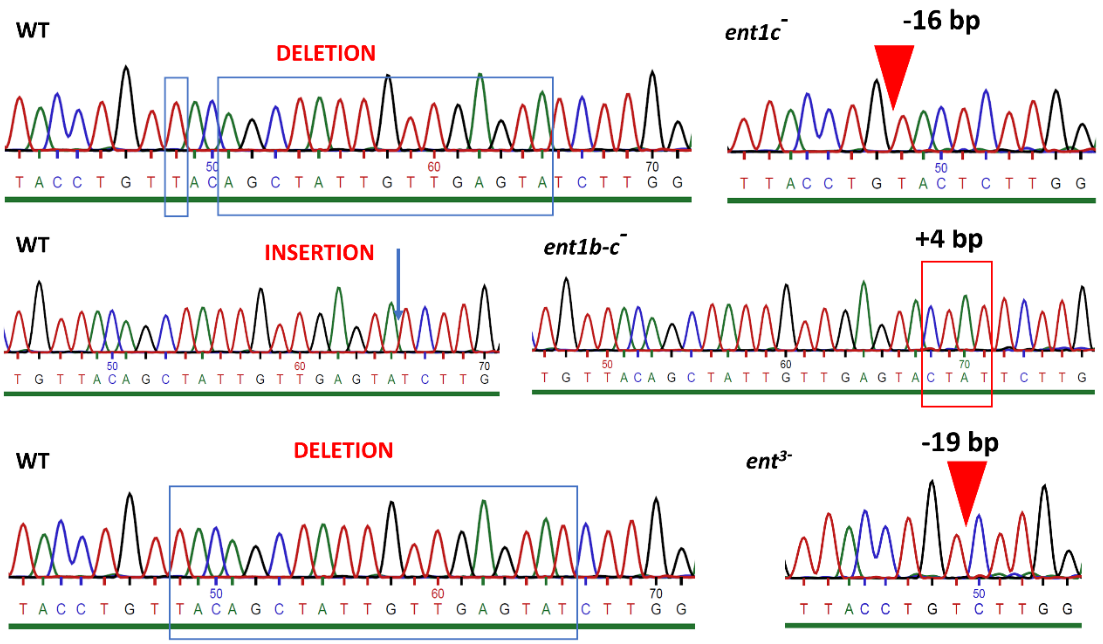

Fig. S4. Generation of *D. discoideum ent1c* null mutants. *Ent1c* gene was disrupted using CRISPR-Cas9 to generate the *ent1c*⁻, *ent1b*-c⁻ or the *ent*$^{3-}$ mutant stains. 16 bp were deleted to generate *ent1c*⁻, 4 bp were added to generate *ent1b*-c⁻ and 19 bp were deleted to generate *ent*$^{3-}$ mutant. Each indel disrupted the open reading frame of the *ent1c* gene and induced several stop codons in the ENT1C protein starting at amino acids 230, 297 and 229 respectively.

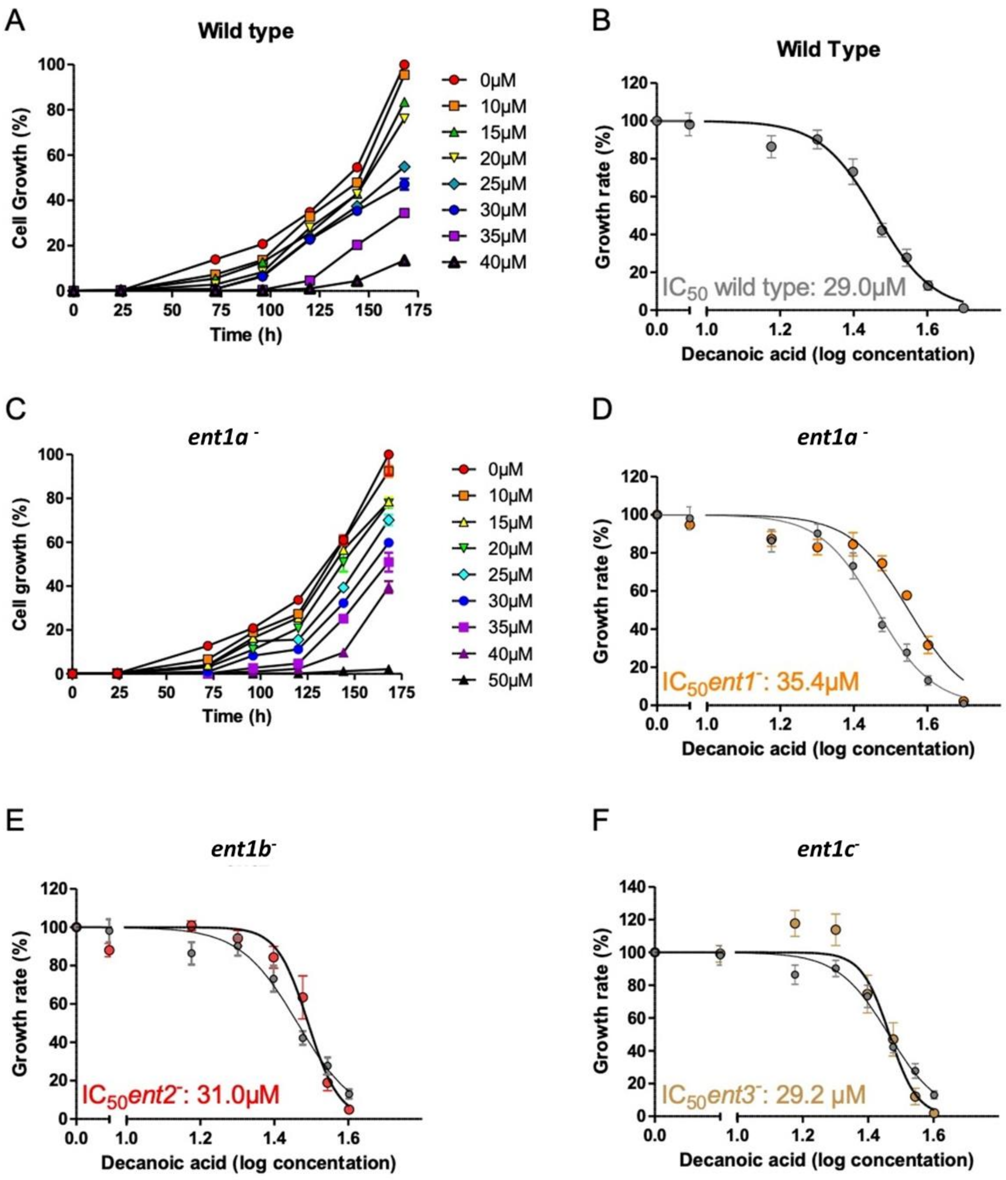

Fig. S5. Cell proliferation effect of decanoic acid on wild type *D. discoideum* and single *ent1* mutants. Wild type (A and B), *ent1a*$^-$ (C and D), *ent1b*$^-$ (E) and *ent1c*$^-$ (F) mutant cells were treated with increasing concentrations of decanoic acid and counted every 24 h for five days. The concentration inhibiting 50% of the growth (IC$_{50,}$) was determined with a non-linear regression of the growth rate of cells during exponential phase plotted against the log concentration of decanoic acid. Growth curves and IC$_{50}$ curves are shown for wild type and *ent1a*$^-$ cells, and only IC$_{50}$ curves are shown for *ent1b*$^-$ and *ent1c*$^-$ cells. Wild type growth rate curve is present on every IC$_{50}$ curve as a comparison. A Mann Whitney test was carried out to compare IC$_{50}$ values and shows that *ent1a*$^-$ mutant cells are significantly more resistant (P=0.036, n=5) to decanoic acid than wild type cells. *ent1c*$^-$ mutant cells do not have any significant modification of IC$_{50}$ but show a resistance for lower decanoic acid concentrations (10-25 µM).

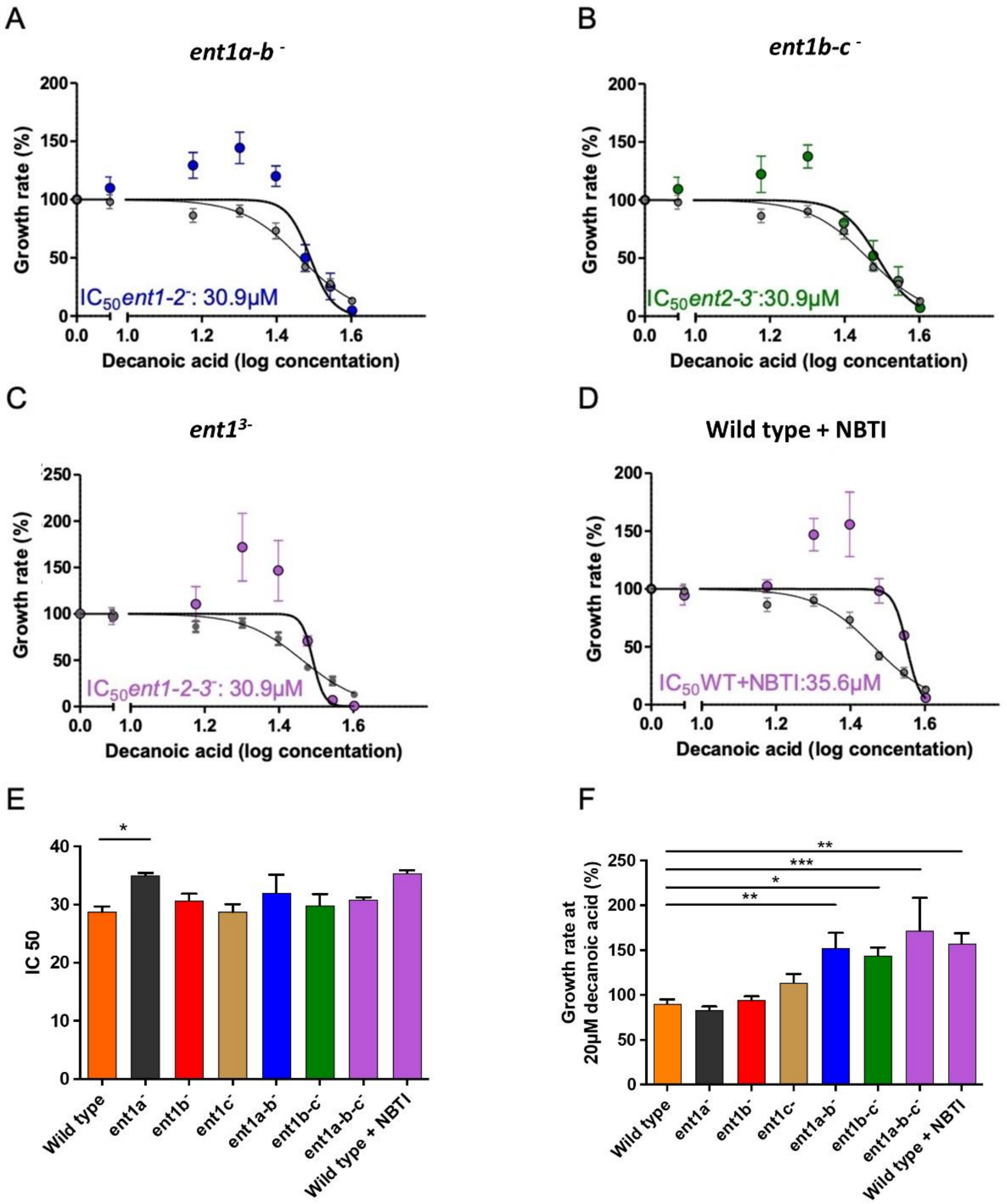

Fig. S6. Cell proliferation effect of decanoic acid on double and triple *ent1* knock out. (A) *ent1a-b*$^-$, (B) *ent1b-c*$^-$, (C) wild type and ENT inhibitor (NBTI), (D) and *ent1a-b-c*$^-$ cells were treated with increasing concentrations of decanoic acid and counted every 24h for five days. The concentration inhibiting 50% of the growth ($IC_{50,}$) was determined with a non-linear regression of the growth rate of cells during exponential phase plotted against the log concentration of decanoic acid. (E) Bar chart of IC_{50} for each mutant shows a significant increase in for *ent1*$^-$ only ($p=0.042$). (F) Bar chart of growth rate at 20 µM was plotted from the dose response curves for each mutant. An ANOVA show a significant increase in growth for all double and triple mutants (*$p<0.05$; **$p<0.01$; ***$p<0.001$). This effect is reproduced by chemical inhibition of ENTs with NBTI.

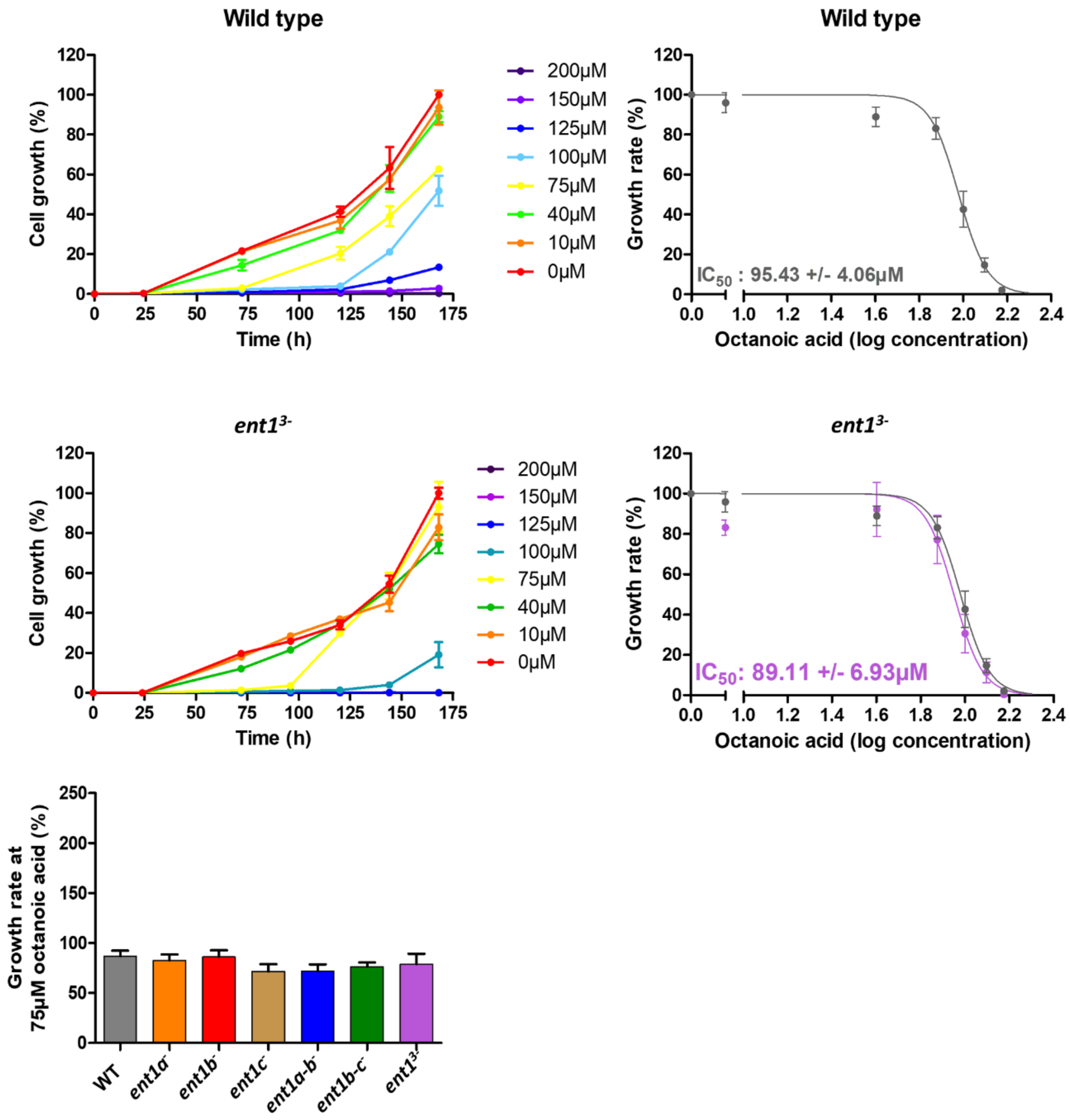

Fig. S7. Cell proliferation effect of octanoic acid on WT and triple *ent1* knock out. (A) *WT,* (B) *ent1*$^{3-}$ cells were treated with increasing concentrations of octanoic acid and counted every 24h for five days. The concentration inhibiting 50% of the growth (IC_{50}) was determined with a non-linear regression of the growth rate of cells during exponential phase plotted against the log concentration of decanoic acid. (C) Bar chart of IC_{50} values for each mutant does not show any significant modification of IC_{50} values.

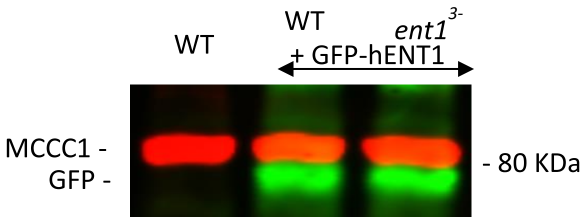

Fig. S8. Western blot analysis of *D. discoideum* wild type and *ent1*$^{3-}$ cells expressing the human GFP-tagged ENT1 (GFP-hENT1) protein. A GFP antibody confirmed expression of the full-length protein (78kDa) (green), absent in wild type cells (loading control, red, 80kDa, MCCC1).Cell proliferation effect of octanoic acid on WT and triple *ent1* knock out.

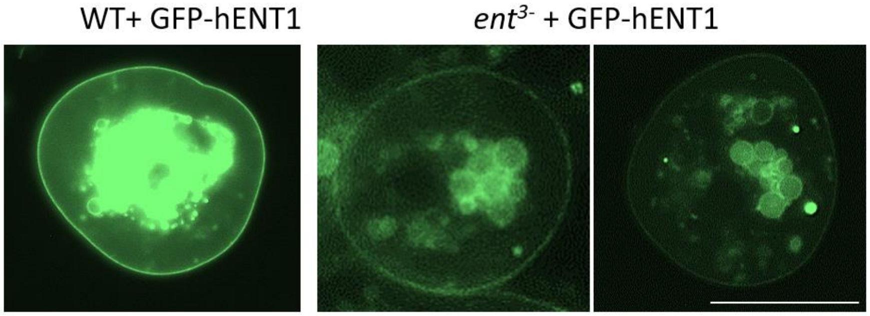

Fig. S9. Further analysis of GFP-hENT1 localisation to the plasma membrane, and endocytic vesicles. Under-agar imaging of wild type and *ent*$^{3-}$ cells expressing GFP-hENT1, where GFP-hENT1 also localises to endocytic vesicle membranes, visualised with the uptake of texas red-labelled dextran from media. Size bar = 10 µM.

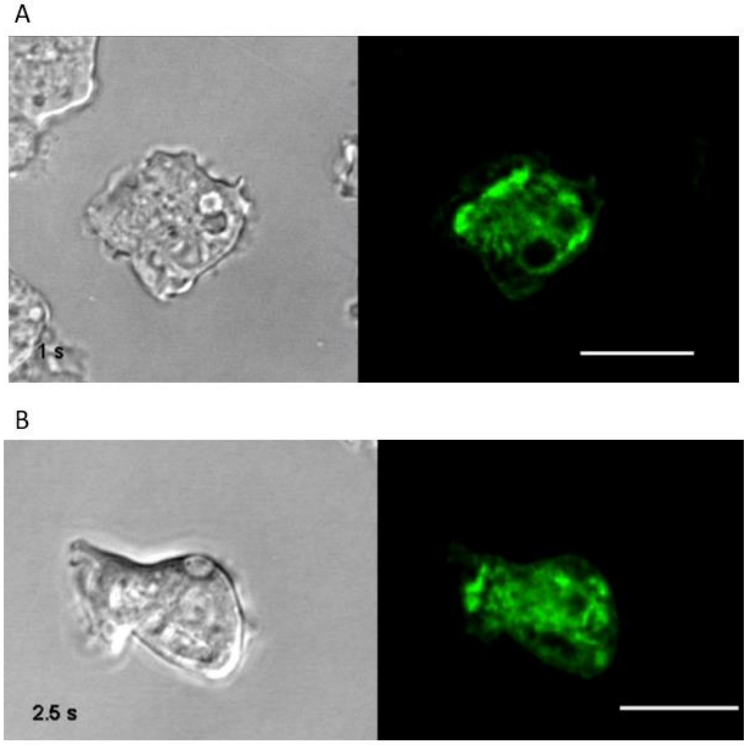

Fig. S10. Still images of time lapse movies of *D. discoideum ent1*$^{3-}$ cells expressing GFP-hENT1. (A, B) Combined brightfield and fluorescent movies showing localisation of GFP-hENT1 (GFP-huEnt1) to plasma membranes, endosomes and contractile vacuoles. Size bar = 10 µM.

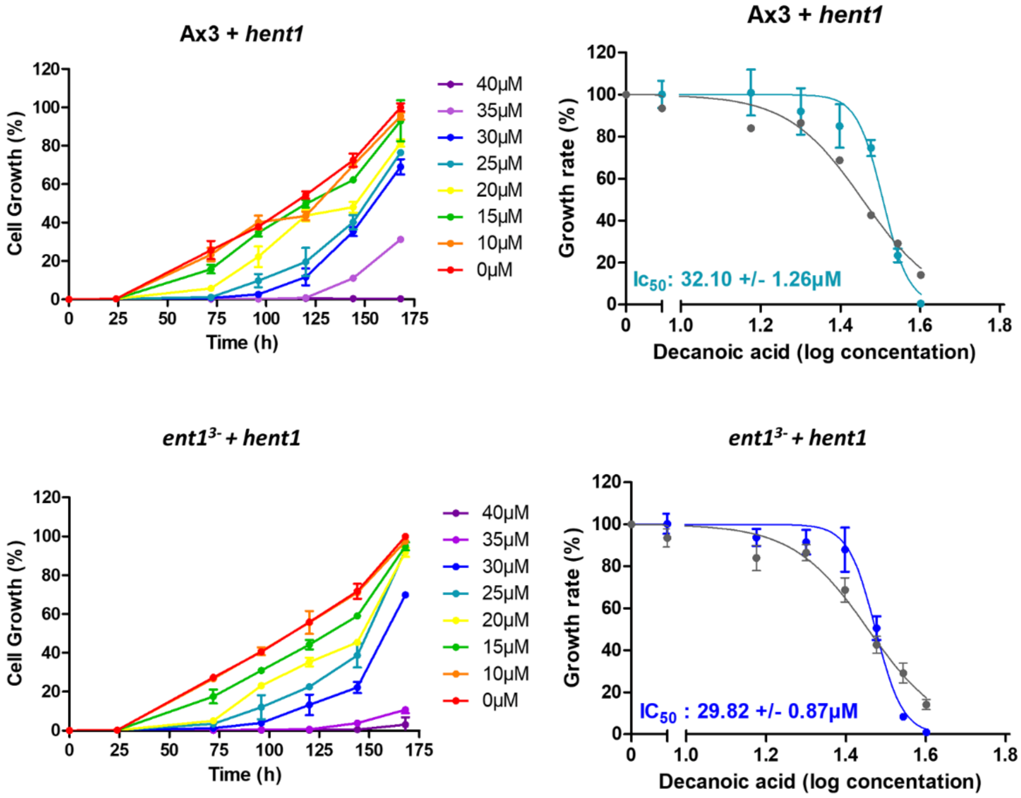

Fig. S11. Cell proliferation analysis of *Dictyostelium* WT and *ent1*$^{3-}$ expressing GFP-hENT1. WT (Ax3) and *ent1*$^{3-}$ cells expressing the human ENT1 (hent1) protein were treated with increasing concentrations of decanoic acid and counted every 24h for five days. The concentration inhibiting 50% of the growth (IC_{50}) was determined with a non-linear regression of the growth rate of cells during exponential phase plotted against the log concentration of decanoic acid.

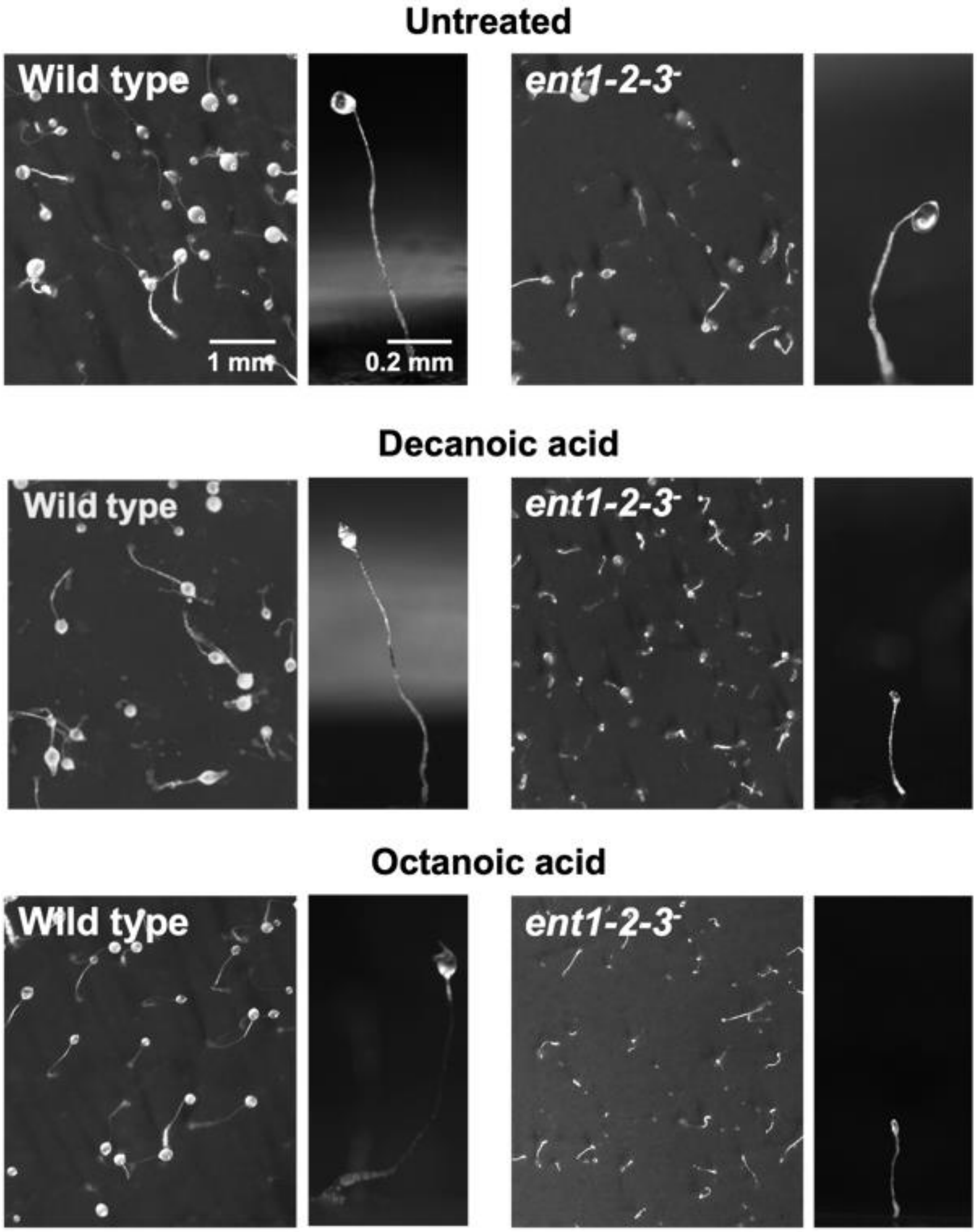

Fig. S12. Development analysis of *D. discoideum* wild type and *ent1*$^{3-}$ cells in the presence of decanoic and octanoic acid. Wild type or *ent1*$^{3-}$ mutant cells were starved and developed for 24 h on nitrocellulose membranes containing solvent only or 100 µM decanoic acid or 200 µM octanoic acid. The morphology of fruiting bodies was recorded from a top- down view of a field of cells or side-on view of a fruiting body. Images show the developmental morphology of wild type and *ents* mutant cells, with the loss of *ents* causing a reduction in fruiting body size without an effect on fruiting body morphology. Both wild type and *ent*$^{3-}$ mutant cells treated with decanoic or octanoic acid formed fruiting bodies with the same morphology as their respective untreated conditions.

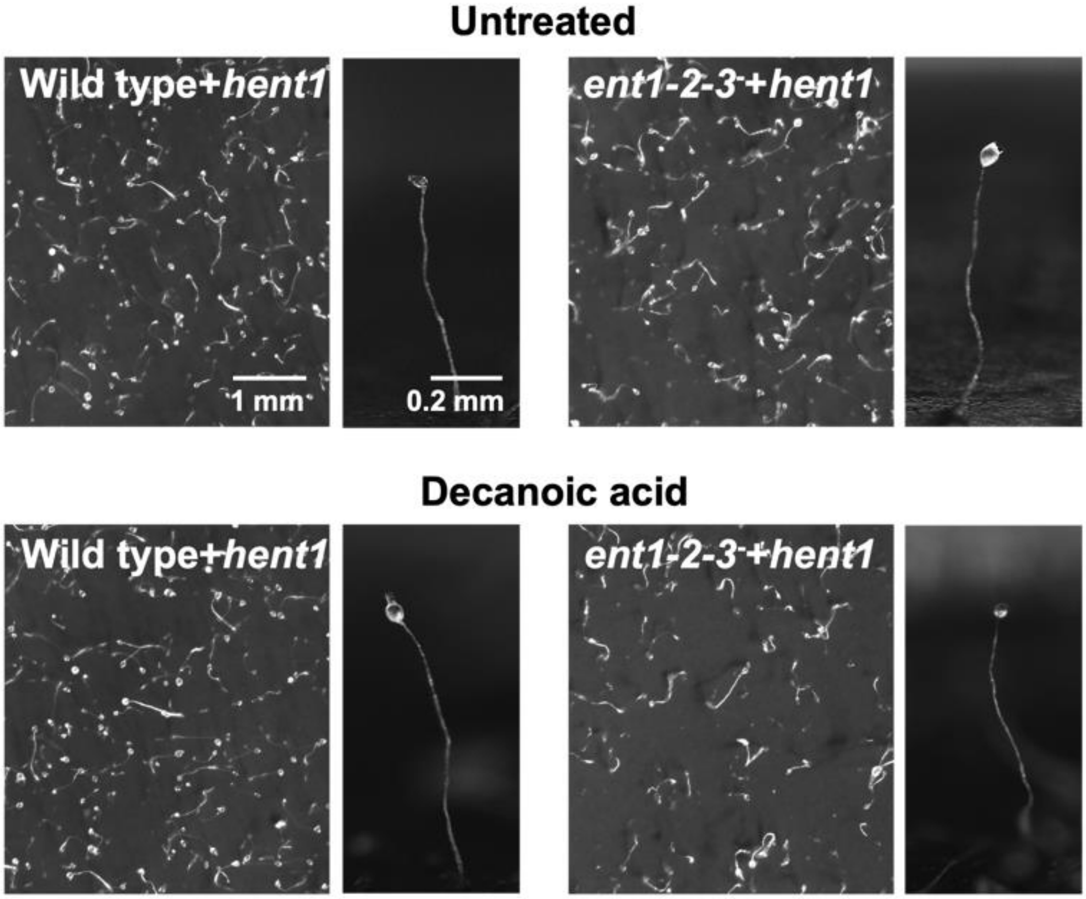

Fig. S13. Development analysis of *D. discoideum* wild type and *ent1*$^{3-}$ cells in the presence of decanoic and octanoic acid. Development analysis of *D. discoideum* wild type and *ent1*$^{3-}$ cells expressing human ENT1 partially restores development. Wild type+hENT1 and *ent1*^{3}-+*hent1* were starve on nitrocellulose membrane for 24h in the presence or absence of 100 µM decanoic acid. The morphology of fruiting bodies was recorded from a top-down view of a field of cells or side-on view of a fruiting body. Images show the developmental morphology of mutant cells forming fruiting bodies with long stalk and round spores in all the conditions (untreated or treated). Development of *ent1*^{3}-+*hent1*was therefore rescued back to wild type developmental phenotype.

Gene name	Gene ID	Gene expression change (log2 (fold change))	p value
FCSB	DDB_G0269474	2.444	4.23E-38
NADDH(1)	DDB_G0290197	1.412	4.21E-22
CSHA	DDB_G0267426	1.797	2.06E-09
ACO(1)	DDB_G0270990	1.572	3.28E-07
ACO1	DDB_G0279159	0.760	1.41E-05
NADDH(2)	DDB_G0270104	0.750	1.44E-04
ACSBG2	DDB_G0270106	0.979	9.82E-04
ACO(2)	DDB_G0289993	0.721	3.39E-03
PATB	DDB_G0282817	1.464	4.91E-03
ALDH(1)	DDB_G0290537	0.637	1.02E-02
ACO(3)	DDB_G0286669	-0.641	1.19E-02
MDHA	DDB_G0290207	0.725	1.56E-02
ALDH(2)	DDB_G0290535	0.805	2.12E-02
GLTA	DDB_G0276965	0.438	3.19E-02
ADA	DDB_G0287371	-0.516	3.77E-02

Table S2. RNAseq analysis of wild type cells following decanoic acid treatment. Cellular energy regulation can be grouped into glycolysis and fatty acid (FA)/β-oxidation, providing acetyl CoA for the tricarboxylic acid cycle (TCA cycle) to provide energy, and to oxidative phosphorylation to provide energy. In wild type cells, significant ($P<0.05$) transcriptional changes of energy metabolism-related genes were observed following decanoic acid (20 µM) 4-day treatment related to fatty acid metabolism, TCA cycle and oxidative phosphorylation. Data is derived from triplicate independent samples. Gene names, provided by Dictybase.org, and matching Gene IDs can be found on the Dictybase web site.

Gene name	Gene ID	Gene expression change (Log2 (fold change))	p value
FCS	DDB_G0269474	1.820	1.15E-89
ACO(1)	DDB_G0270990	0.929	1.37E-17
ACAT	DDB_G0271544	-0.603	2.07E-08
ACO(2)	DDB_G0289993	0.550	5.08E-07
ALDH(1)	DDB_G0276821	0.389	4.12E-06
ACAA1	DDB_G0274339	0.334	0.000578
ACSBG	DDB_G0270106	0.357	0.002544
ACAD8	DDB_G0288647	0.283	0.007931
CSHA	DDB_G0267426	0.997	7.06E-26
SCSC	DDB_G0271842	0.487	1.44E-08
MDHA	DDB_G0290207	0.470	7.3E-07
ACO1	DDB_G0279159	0.398	4.36E-06
MDHB	DDB_G0292600	0.380	7E-06
IDHA	DDB_G0271344	0.339	2.67E-05
SCSA	DDB_G0289325	0.380	2.87E-05
MDHC	DDB_G0280255	0.284	0.003242
PDHB	DDB_G0276417	0.254	0.004217
FUMH	DDB_G0280495	0.233	0.008314
GLTA	DDB_G0276965	0.311	0.01002
SDHC	DDB_G0275115	0.221	0.027129
ODHB	DDB_G0275029	0.222	0.043245
NUOR	DDB_G0290197	0.838	1.32E-13
PATB	DDB_G0282817	0.657	9.49E-07
ATP5O	DDB_G0283283	0.324	0.000386
COX11	DDB_G0289353	-0.527	0.000812
VATE	DDB_G0275701	0.289	0.001192
VATA	DDB_G0287127	0.264	0.001369
ATP5D	DDB_G0269038	0.303	0.001452
NDUFV1	DDB_G0288875	0.306	0.002626
NDUFA12	DDB_G0285783	0.382	0.003293
UQCRB	DDB_G0286171	0.298	0.003764
ATP5C1	DDB_G0292306	0.257	0.004982
CYC1	DDB_G0292594	0.246	0.005119
ATP5B	DDB_G0269916	0.238	0.006476
NDUFAB1	DDB_G0291866	0.281	0.006566
VATP	DDB_G0274381	0.287	0.007184
NDUFA5	DDB_G0284799	0.386	0.009539
NDUAD	DDB_G0272394	0.327	0.016536
VATG	DDB_G0274311	0.334	0.017175
NDUA2	DDB_G0272476	-0.315	0.019458
COXA	DDB_G0281549	-0.225	0.0213
VATG	DDB_G0277971	0.332	0.021653
UCR	DDB_G0284947	0.212	0.022463
COX10	DDB_G0288169	-0.279	0.023898
NDUFS8	DDB_G0277231	0.230	0.038986
DGK	DDB_G0280843	-0.806	0.040718
ADA	DDB_G0287371	-0.594	5E-11

Table S3. RNAseq analysis of wild type cells treated with ENT1 inhibitor following decanoic acid treatment. Cellular energy regulation can be grouped into glycolysis and fatty acid (FA)/β-oxidation, providing acetyl CoA for the tricarboxylic acid cycle (TCA cycle) to provide energy, and to oxidative phosphorylation to provide energy. In wild type cells treated with ENT1 inhibitor (10 µM NBTI), an increased number of significant ($P<0.05$) transcriptional changes of energy metabolism-related genes were observed following decanoic acid (20 µM) 4day treatment related to fatty acid metabolism, TCA cycle and oxidative phosphorylation. Data is derived from triplicate independent samples. Gene names, provided by Dictybase.org, and matching Gene IDs can be found on the Dictybase web site.

Supplementary Movie S1. Time lapse imaging of *D. discoideum ent1*$^{3-}$ cells expressing GFP-hENT1. Combined brightfield and fluorescent movies showing localisation of GFP-hENT1 (GFP-huEnt1) to plasma membranes, endosomes and contractile vacuoles. Size bar = 10 µM

Supplementary Movie S2. Time lapse imaging of *D. discoideum ent1*$^{3-}$ cells expressing GFP-hENT1. Combined brightfield and fluorescent movies showing localisation of GFP-hENT1 (GFP-huEnt1) to plasma membranes, endosomes and contractile vacuoles. Size bar = 10 µM

SI References

1. M. Fukuzawa, N. V. Zhukovskaya, Y. Yamada, T. Araki, J. G. Williams, Regulation of Dictyostelium prestalk-specific gene expression by a SHAQKY family MYB transcription factor. *Development* **133**, 1715-1724 (2006).
2. H. J. R. Fernandes *et al.*, Mitochondrial and Endoplasmic Reticulum Stress Trigger Triglyceride Accumulation in Models of Parkinson's Disease Independent of Mutations in MAPT. *Metabolites* **13** (2023).
3. E. C. Warren *et al.*, Decanoic acid inhibits mTORC1 activity independent of glucose and insulin signaling. *Proc Natl Acad Sci U S A* **117**, 23617-23625 (2020).
4. A. J. Davidson, J. S. King, R. H. Insall, The use of streptavidin conjugates as immunoblot loading controls and mitochondrial markers for use with Dictyostelium discoideum. *Biotechniques* **55**, 39-41 (2013).
5. R. D. Rosengarten, B. Santhanam, J. Kokosar, G. Shaulsky, The Long Noncoding RNA Transcriptome of Dictyostelium discoideum Development. *G3 (Bethesda)* **7**, 387-398 (2017).
